# Hyaluromycin, a New Hyaluronidase Inhibitor of Polyketide Origin from Marine *Streptomyces* sp.

**DOI:** 10.3390/md12010491

**Published:** 2014-01-21

**Authors:** Enjuro Harunari, Chiaki Imada, Yasuhiro Igarashi, Takao Fukuda, Takeshi Terahara, Takeshi Kobayashi

**Affiliations:** 1 Graduate School of Marine Science and Technology, Tokyo University of Marine Science and Technology, 4-5-7 Konan, Minato-ku, Tokyo 108-8477, Japan; E-Mails: imada@kaiyodai.ac.jp (C.I.); terahara@kaiyodai.ac.jp (T.T.); takeshik@kaiyodai.ac.jp (T.K.); 2 Biotechnology Research Center, Toyama Prefectural University, 5180 Kurokawa, Imizu, Toyama 939-0398, Japan; E-Mails: yas@pu-toyama.ac.jp (Y.I.); z02113@st.pu-toyama.ac.jp (T.F.)

**Keywords:** rubromycin, hyaluronidase inhibitor, marine actinomycetes, *Streptomyces*, 2-amino-3-hydroxycyclopent-2-enone

## Abstract

Hyaluromycin (**1**), a new member of the rubromycin family of antibiotics, was isolated from the culture extract of a marine-derived *Streptomyces* sp. as a HAase inhibitor on the basis of HAase activity screening. The structure of **1** was elucidated through the interpretation of NMR data for the compound and its 3″-*O*-methyl derivative in combination with an incorporation experiment with [1,2-^13^C_2_]acetate. The compound’s absolute configuration was determined by the comparison of its circular dichroism (CD) spectrum with those of other rubromycins. Hyaluromycin (**1**) consists of a γ-rubromycin core structure possessing a 2-amino-3-hydroxycyclopent-2-enone (C_5_N) unit as an amide substituent of the carboxyl function; both structural units have been reported only from actinomycetes. Hyaluromycin (**1**) displayed approximately 25-fold more potent hyaluronidase inhibitory activity against hyaluronidase than did glycyrrhizin, a known inhibitor of plant origin.

## 1. Introduction

Hyaluronidase (HAase)—an endoglycosidase—hydrolyzes hyaluronic acid (HA), the only non-sulfated glycosaminoglycan that is not attached to a core protein, which consists of a repeating unit of D-glucuronic acid and *N*-acetyl glucosamine. HA was synthesized by HA synthases which polymerize HA on the intracellular membrane surface. The HA polymers are extruded onto the glycocalyx or into the extra cellular matrix (ECM). HA exists mainly in the skin, primarily in the dermis, of mammalians and is degraded by various different HAases in the somatic cells in a step-by-step manner. HAase is found in a number of organisms, including mammals, bacteria (*Streptomyces* [1], *Streptococcus* [2]) and bacteriophages [3], as well as in the venom of terrestrial (bees [4], hornets [5], scorpions [6], snakes [7], lizards [8]) and marine (krill [9], lobster [10], fishes [11]) animals. One HAase is an acid-active enzyme in the mammalian circulatory system. Three types of eukaryotic HAase exist: Neutral-active endo-β-*N*-glucosaminidase, acidic-active endo-β-*N*-glucosaminidase, and endo-β-glucuronidase.

HAs have several functions in inflammation [12,13], immunity oncogenesis, *etc*., largely depending on the molecular size of the polymer. Low-molecular weight HAs stimulate angiogenesis [14] and the induction of chemokines [15] and cytokines [16,17,18], while high-molecular weight HAs suppress these phenomena [13,15,19]. Recently, extremely high-molecular weight HAs (6–12 MDa) were found in naked mole-rat (*Heterocephalus glaber*) fibroblasts [20]; these compounds were more than five times larger than human and mouse HAs (0.5–3 MDa) [21]. Although the naked mole rat is known as an exceptionally long-lived rat (40/86, many of which were alive after 24 years) [22], surprisingly, neoplasms have never been found in the rat [22,23]. However, in the rats with HA synthase knockdown or HAase overexpression, tumor formation was observed [21].

HAase is some molecular target of anti-inflammatory and anti-allergic drugs. For example, anti-allergic agents such as disodium cromoglycate (DSCG) and tranilast and anti-inflammatory agents, like glycyrrhizin, demonstrate HAase inhibitory activity [24,25,26]. Further, the compound 48/80, a well-known histamine-releasing agent [27], also activates HAase [27]. Therefore, HAase inhibitors may become candidate compounds for anti-inflammatory drugs. In a previous study, various types of HAase inhibitors were reported: Proteins, glycosaminoglycans, polysaccharides, fatty acids, alkaloids, flavonoids, terpenoids, antioxidants, polyphenols, antibiotics, antinematodes, lanostanoids, synthetic organic compounds, glycosides and saponins [28]. However, very few studies have been reported on a screening of HAase inhibitor from microbial and marine-derived compounds. Also, the screening from actinomycetes has not been reported to the best of our knowledge.

**Figure 1:**
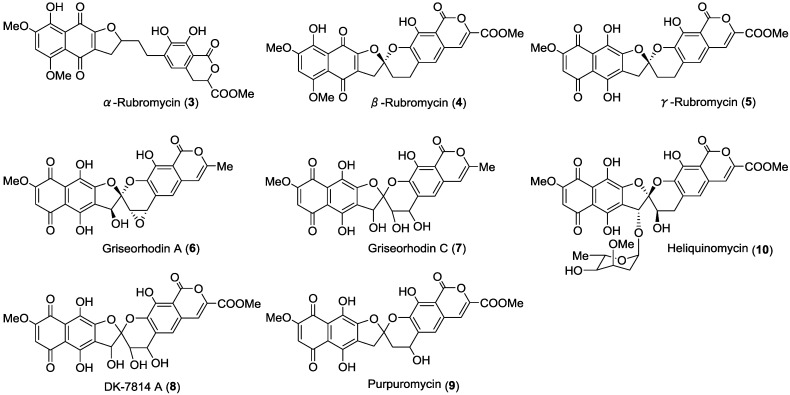
Natural rubromycins.

The rubromycin family of antibiotics consists of reddish polyketide pigments produced by some groups of actinomycetes (*Streptomyces*, *Dactylosporangium* and *Actinoplanes*). These compounds possess a naphthoquinone ring and an isocoumarin ring connected through a 5,6-spiroketal system. The rubromycins α (**3**), β (**4**), γ (**5**) [29,30], δ and 3′-hydroxy-β-rubromycin [31]; the griseorhodins A (**6**), C (**7**) and G [32]; the DK-7814s A (**8**), B and C [33]; purpuromycin (**9**) [34] and heliquinomycin (**10**) [35] are compounds that are structurally related to the rubromycin family (Figure 1). Rubromycins show inhibitory activity against human telomerase and the reverse transcriptase of human immunodeficiency virus-1 [36]. α-Rubromycin (**3**) is the only compound in this family that lacks the spiroketal structure, and it displays much lower activity against telomerase and HIV reverse transcriptase-1 than its spiroketal congeners [36]; this suggests that the spiroketal system acts as a pharmacophore for telomerase and HIV inhibition. Further, several structurally related analogues of rubromycins also inhibit telomerase [37]. Heliquinomycin, a glycosylated derivative of the rubromycins, bears a cymarose moiety, and exhibits a DNA helicase inhibitory activity against a wide range of cancer cells [35].

**Figure 2:**
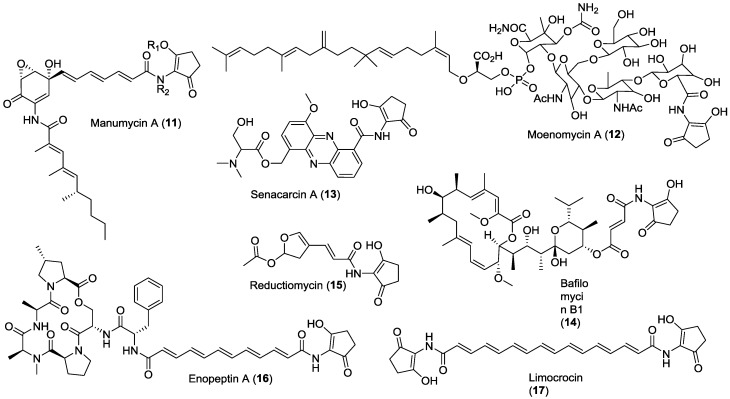
Natural products containing the C_5_N substructure.

**Figure 3:**
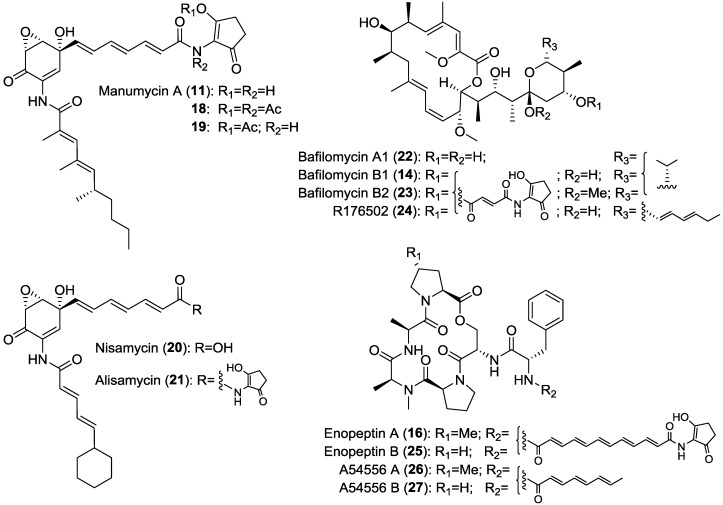
Families of compounds containing the C_5_N substructure.

The 2-amino-3-hydroxycyclopent-2-enone (C_5_N unit) has been found to be a partial structure of polyketides from actinomycetes (Figure 2). Most of the C_5_N unit-containing metabolites were isolated from *Streptomyces* and the others were isolated from *Micromonospora* (micromonospolide [38], R176502 [39]), *Kitasatospora* (bafilomycin B1 (**14**) [40], Sch 725424 [41]) and *Amycolatopsis* (ECO-0501 [42]). Compounds containing the C_5_N unit include diverse structural types such as manumycins [43], moenomycins [44], bafilomycins [40], enopeptins [45], senacarcins [46], limocrocin (**17**) [47], reductiline [48], reductiomycins [49], virustomycins [50], ECO-02301 [51], ECO-0501 [42], and 2880-II [52]. The molecular weights of these compounds range from 300 to 1500 daltons. Although 2-acetamino-3-hydroxycyclopent-2-enone itself developed no microbial activity, in the case of manymycin A (**11**), acetylation of 3″-OH (**18**) and 2″-NH (**19**) in the C_5_N unit led to a decrease of the biological activity [53]. Nisamycin (**20**), **11** that lacks the C_5_N unit, displayed six-fold more active antimicrobial activity than alisamycin (**21**), the analog that contains the C_5_N unit [54]. For the bafilomycins (**14**, **22** and **23**) and enopeptins (**16**, **25**, **26** and **27**), no significant differences were observed in the microbial activity of compounds containing or lacking the C_5_N unit [40,55]. Three bafilomycins compounds, bafilomycin A1 (**22**) (lacking the C_5_N unit), **14** (containing the C_5_N unit) and R176502 (**24**) (**14** analog) exhibited similar potency for the inhibition of tumor cell proliferation [39]. These results suggest that the 2-amino-3-hydroxycyclopent-2-enone sub-structure is not related to the biological activity of these compounds (Figure 3).

**Figure 4:**
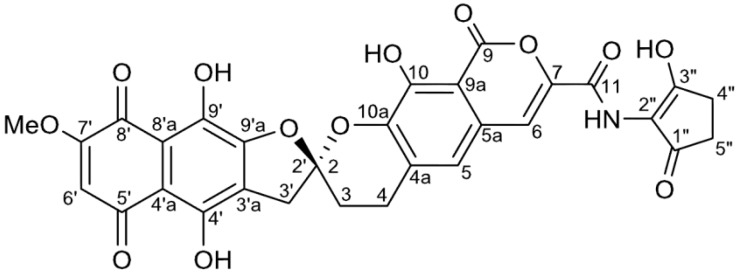
Structure of hyaluromycin (**1**).

The objective of this study is to obtain a new type of HAase inhibitor as an anti-inflammatory candidate compound from marine derived-actinomycetes. In this paper, we report the isolation, structural elucidation and bioactivity of hyaluromycin (**1**) (Figure 4), a new member of rubromycin family, from the culture extract of the *Streptomyces* sp. strain MB-PO13 isolated from marine sea squirt (*Molgula manhattensis*). This strain was selected from approximately 1,000 marine organism-derived actinomycete strains through the screening of anti-inflammatory compounds on the basis of HAase inhibitory activity. In the last section of this paper, we report the results of the assays on the HAase inhibition of **1**, derivative **1**, rubromycins and glycyrrhizin, a known HAase inhibitor.

## 2. Results and Discussion

The producing strain MB-PO13 was cultured in A-3M medium at 30 °C for seven days, and the entire culture broth was extracted with EtOAc at pH 3. The extract was fractionated by reversed-phase column chromatography, followed by HPLC purification on a C18 column, to yield (**1**) as an optically active, red amorphous powder ([α]^25^_D_ −168, DMSO). The molecular formula of C_30_H_21_NO_13_ was confirmed by high-resolution ESITOFMS data showing a pseudomolecular ion [M + H]^+^ at *m*/*z* 604.1091. The IR spectrum indicated the presence of hydroxyl (3357 cm^−1^) and carbonyl (1693 cm^−1^) functional groups. The UV spectrum showed absorption maxima at 307, 352, 368 and 506 nm similar to those of the rubromycin class of antibiotics [29,30,31,32,33,34,35].

**Figure 5:**
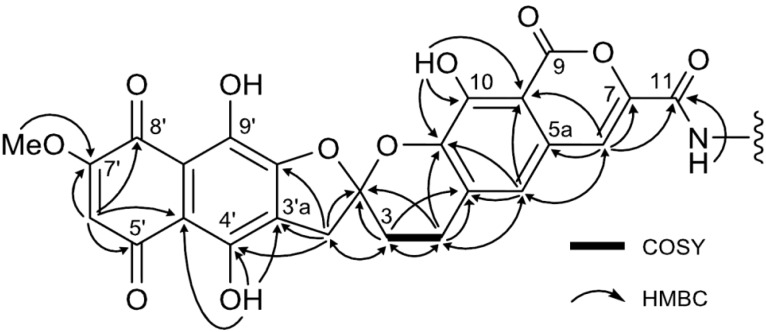
^1^H-^1^H COSY and HMBC correlations of compound **1**.

The ^1^H NMR spectrum of **1** measured in DMSO-*d*_6_ indicated the presence of one methoxy (δ_H_ 3.89), three methine (δ_H_ 6.41, 7.25 and 7.52) and four exchangeable (δ_H_ 9.44, 10.67, 11.90 and 13.14) protons. In the ^13^C NMR spectrum, all of the 25 carbons assignable to γ-rubromycin core were detected. Comparison with the MS data showed that five carbon atoms were lacking [31,56,57]. The ^1^H-^1^H COSY spectrum established only one H-3/H-4 spin system. Further HSQC and HMBC analysis allowed the assignment most of the ^13^C signals except for C-9, C-8′a and C-9′ (Figure 5). An exchangeable proton at δ_H_ 9.44 showed a correlation with C-11, suggesting that this proton could be an amide proton.

**Figure 6:**
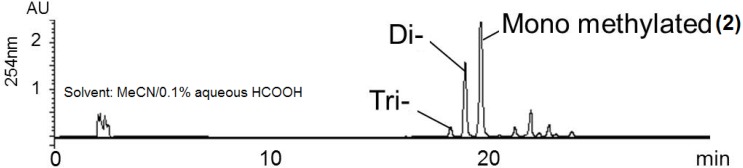
HPLC chromatogram of methylated derivatives of **1**.

The NMR data and UV spectrum of **1** strongly indicated the presence of a γ-rubromycin (5) skeleton, but three carbons remained unassigned. Additionally, five further carbons were not detected in the ^13^C NMR spectrum. To establish the aromatic polyketide structure, a feeding experiment was conducted using [1,2-^13^C_2_]acetate to obtain ^13^C-enriched **1** for a 2D-INADEQUATE experiment. However, in the INADEQUATE spectrum, cross peaks were not observed because of the low concentration of **1** in NMR solvents. To improve its solubility, an *O*-methylation reaction of ^13^C-labeled **1** was carried out using an excess of methyl iodide and 1,8-diazabicyclo[5,4,0]-7-undecene (DBU) in MeCN/acetone. The reaction proceeded smoothly at 50 °C, and the starting material (**1**) was consumed within 1 h. The crude extract contained a mixture of three *O*-methylated adducts, whose structures were deduced from LC/MS analysis, to be in order of elution, the mono-, di- and trimethyl derivatives of **1** (Figure 6). The monomethyl derivative (**2**) was purified by preparative HPLC, and its structure was determined as follows.

**Figure 7:**
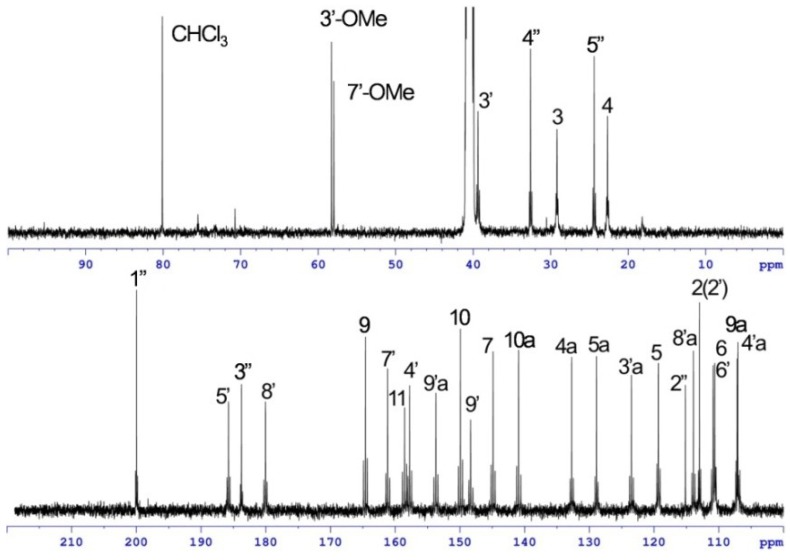
^13^C NMR spectrum of [1,2-^13^C_2_]acetate-labeled **2**.

**Figure 8:**
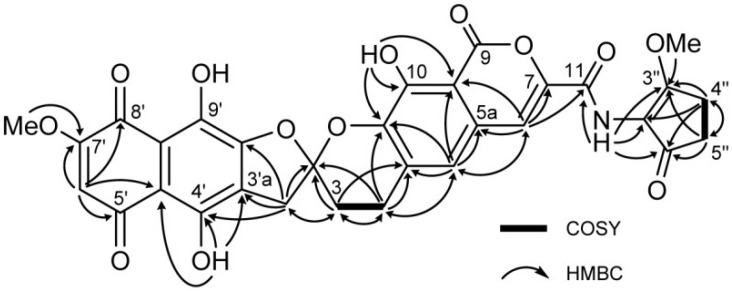
^1^H-^1^H COSY and HMBC correlations of [1,2-^13^C_2_]acetate-labeled **2**.

**Figure 9:**
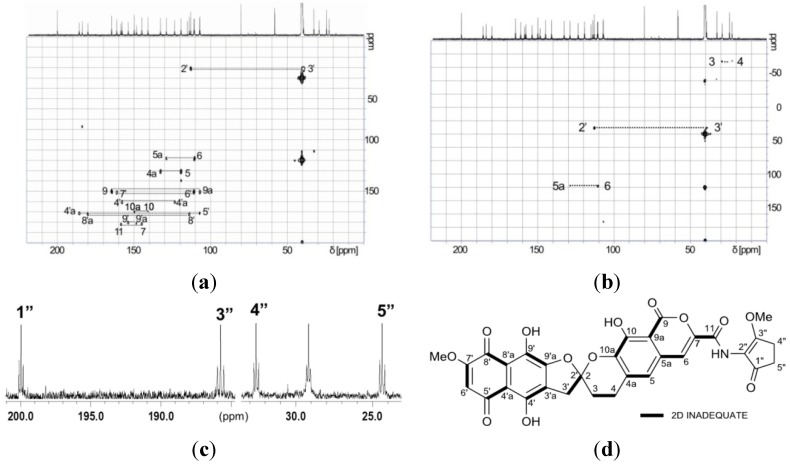
^13^C-^13^C couplings observed in 2D INADEQUATE (**a**,**b**) and ^13^C (**c**) NMR spectra of [1,2-^13^C_2_]acetate-labeled **2**. The coupling of C-1″/C-5″ and C-3″/C-4″ were only observed in the ^13^C NMR spectrum (**c**). (**a**) Optimized for ^1^*J*_cc_ = 50 Hz; (**b**) Optimized for ^1^*J*_cc_ = 35 Hz; (**c**) ^13^C NMR spectra; (**d**) Observed in 2D INADEQUATE.

The solubility of [1,2-^13^C_2_]acetate-labeled **2** in DMSO-*d*_6_ was much improved, allowing a high-quality ^13^C NMR spectrum to be obtained in which 31 discrete resonances could be observed (Figure 7). Of these resonances, 25 carbons were readily assigned to the rubromycin core on the basis of HMBC correlations (Figure 8, Table 1). The three carbons C-9, C-8′a and C-9′ had no HMBC correlations but were assigned on the basis of the INADEQUATE experiments. In the 2D-INADEQUATE spectrum of [1,2-^13^C_2_]acetate-labeled **2**, with the parameters optimized for ^1^*J*_CC_ 50 Hz, cross peaks were observed for all of the carbons of the rubromycin core structure: C-4a/C-5, C-5a/C-6, C-7/C-11, C-9/C-9a, C-10/C-10a, C-2′/C-3′, C-3′a/C-4′, C-4′a/C-5′, C-6′/C-7′, C-8′/C-8′a and C-9′/C-9′a, with the exception of C-3/C-4 (Figure 9a). Because the coupling constant for C-3/C-4 read from the ^13^C NMR spectrum (Figure 7) was smaller (31.1 Hz), the INADEQUATE spectrum was measured with a parameter set optimized for ^1^*J*_CC_ 35 Hz, which indicated a cross peak between C-3 and C-4 (Figure 9b), establishing the complete ^13^C NMR assignment for the rubromycin core of **2** (Figure 9d). Although cross peaks were not observed for C-1″, C-2″, C-3″, C-4″, and C-5″, the coupling constants ^1^*J*_CC_ for C-1″/C-5″ (38.3 Hz) and C-3″/C-4″ (40.2 Hz) established these carbons as belong to two separate acetate units (Figure 9c). The incorporation patterns of [1,2-^13^C_2_]acetate in the C_5_N unit were consistent with those obtained for manumycin and asukamycin [58].

**Table 1:** NMR data for hyaluromycin (**1**) and [1,2-^13^C_2_]acetate-labeled **2** in DMSO-*d*_6_.

Position	1				[1,2-^13^C_2_]Acetate-labeled 2		
	δ_C_^a^, Type	δ_H_, Mult. (*J* in Hz) ^b^	HMBC ^c^	δ_C_^a^, Type	^1^*J*_CC_ (Hz), Mult.	δ_H_, Mult. (*J* in Hz) ^b^	HMBC ^c^
2 (2′)	113.0, qC			113.0, qC	42.4, dd		
3	29.1, CH_2_	2.37, m; 2.56, m	2, 4a	29.2, CH_2_	30.8, dd	2.38–2.60, m	2, 4a
4	22.7, CH_2_	3.06, m; 3.17, m	2, 3, 4a, 5, 10a,	22.7, CH_2_	31.4, dd	3.07, m; 3.19, m	2, 3, 4a, 5, 10a
4a	132.8, qC			132.7, qC	59.8, dd		
5	119.5, CH	7.25, s	4a, 5a, 6, 9a, 10a	119.3, CH	59.9, dd	7.24, s	4a, 5a, 6, 9a, 10a
5a	128.9, qC			128.9, qC	54.6, dd		
6	110.9, CH	7.52, s	5, 5a, 7, 9a, 11	110.6, CH	56.0, dd	7.50, s	5, 5a, 7, 9a, 11
7	144.7, qC			144.9, qC	78.6, dd		
9	164.4, qC			164.6, qC	72.5, dd		
9a	107.0, qC			106.9, qC	76.3, dd		
10	150.0, qC			149.9, qC	79.1, dd		
10a	141.1, qC			140.9, qC	78.9, dd		
11	158.8, qC			158.5, qC	78.4, dd		
2′ (2)	113.0, qC			113.0, qC	42.4, dd		
3′	39.4, CH_2_	3.55, d (18.0)	3, 2′, 3′a, 4′, 9′a	39.4, CH_2_	42.8, dd	3.49, d (17.9)	3, 2′, 3′a, 4′, 9′a
		3.48, d (18.0)				3.63, d (17.9)	
3′a	123.6, qC			123.5, qC	71.6, dd		
4′	157.8, qC			157.8, qC	71.4, dd		
4′a	107.2, qC			107.2, qC	56.5, dd		
5′	185.9, qC			185.8, qC	38.4, dd		
6′	110.9, CH	6.41, s	4′a, 5′, 7′, 8′	110.9, CH	65.5, dd	6.42, s	4′a, 5′, 7′, 8′
7′	161.2, qC			161.2, qC	71.3, dd		
8′	180.2, qC			180.1, qC	59.1, dd		
8′a	114.0, qC			113.9, qC	59.1, dd		
9′	148.3, qC			148.3, qC	73.9, dd		
9′a	153.7, qC			153.7, qC	74.8, dd		
7′-OMe	58.0, CH_3_	3.89, s	7′	58.0, CH_3_		3.91, s	7′
1″	n.d. ^d^			200.0, qC	38.4, dd		
2″	113.9, qC			115.1, qC			
3″	n.d. ^d^			183.8, qC	39.8, dd		
4″	n.d. ^d^			32.6, CH_2_	40.5, dd	2.41, m	1″, 3″, 4″
5″	n.d. ^d^			24.4, CH_2_	38.3, dd	2.83, dd (3.7, 3.7)	1″, 2″, 3″, 5″
3″-OMe				58.3, CH_3_		4.01, s	3″
10-OH		10.67, s	9a, 10, 10a			10.71, s	9a, 10, 10a
4′-OH		13.14, s	3′a, 4′, 4′a			11.88, s	3′a, 4′, 4′a
9′-OH		11.90, s				13.15, s	
11-NH		9.44, s	11			9.44, s	11, 1″, 2″, 3″

^a^ Recorded at 125 MHz; ^b^ Recorded at 500 MHz; ^c^ Correlation are from proton(s) to carbon; ^d^ Not detected.

The remaining six carbons were attributed to the methoxycyclopentenone moiety based on the 2D-NMR analytical data. A COSY cross peak between H-4″ and H-5″ established a two-carbon fragment consisting of two methylene groups. H-4″ was correlated with the three sp^2^ trisubstituted carbons C-1″ (δ_C_ 200.0), C-2″ (δ_C_ 115.1), and C-3″ (δ_C_ 183.8) and H-5″ to C-1″. An HMBC correlation between the protons of OMe-3″ and the carbon C-3″ and the chemical shifts of the aforementioned three carbons established the presence of a cyclopent-2-enone bearing a methoxy substitution at the 3-position. This six-carbon unit was connected to C-11 through an amide linkage on the basis of the HMBC correlations of an amide proton (δ_H_ 9.44) with C-1″, C-2″, and C-3″, finally providing the full planar structure of **2**. ^1^H and ^13^C NMR resonances for the 3-hydroxycyclopent-2-enone subunit of **1** were not detected. This result could be attributed to the keto-enol tautomerization of the 1,3-diketone structure. All carbons for the cyclopentenone unit were detected in the methylated derivative **2**, in which tautomerization does not occur. Similar observations have been reported for several other compounds containing the same 1,3-diketo substructure [40,59,60].

**Figure 10:**
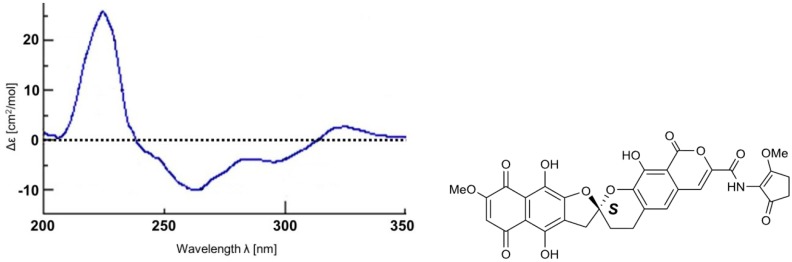
CD spectrum and absolute configuration of **2**.

The absolute configuration of the spiro carbon C-2 (C-2′) of **2** was determined from circular dichroism (CD) data. In the CD spectrum of **2**, two characteristic Cotton effects were observed (Figure 9), a positive one at 224 nm (Δε = +25.7) and a negative one at 262 nm (Δε = −10.0). These results are in agreement with those previously obtained for β-rubromycin (**4**), γ-rubromycin (**5**) and griseorhodin A (**6**) [61,62]. Therefore, the absolute configuration of **2** was determined to be *S* (Figure 10). To our knowledge, the only member of this family of compounds possessing an *R* configuration of the spiro center is heliquinomycin (**10**), whose absolute configuration was deduced from X-ray analysis and which possesses Cotton effects opposite to those of the above molecules [34].

Hyaluromycin (**1**) displayed 25-fold more potent inhibitory activity against HAase from bovine testes with an IC_50_ value of 14 μM, than did glycyrrhizin (IC_50_ = 340 μM), a well-known plant terpenoid [26]. Interestingly, β-rubromycin (**4**) and γ-rubromycin (**5**), which lacks the aminocylopentenone unit, showed no inhibitory activity in the concentration range from 0.013% to 0.5%. More noteworthy is that the derivative **2**, in which the enol hydroxyl group in the cyclopentane unit is protected as a methyl ether, showed no inhibitory activity in the concentration range from 0.013% to 0.5%. These results suggest that the 2-amino-3-hydroxycyclopent-2-enone subunit, and possibly its tautomeric structure, play an essential role in hyaluronidase inhibition (Table 2).

**Table 2:** HAase inhibition (%) of **1**, **2**, β-rubromycin (**4**), γ-rubromycin (**5**) and glycyrrhizin.

Compound	0.0031	0.0063	0.013	0.025	0.050	0.10	0.25	0.50	1.0	2.0	(%)
**1**	7.1	33.5	70.5	94.2	94.9	96.7					
**2**			0	0	0	0	0	0			
β-Rubromycin (**4**)			0	0	0	0	0	0			
γ-Rubromycin (**5**)			0	0	0	0	0	0			
Glycyrrhizin					10.6	20.0	37.9	87.6	99.1	99.2	

## 3. Experimental Section

### 3.1. General Experimental Procedures

Sodium [1,2-^13^C_2_]acetate was purchased from Sigma-Aldrich Co. LLC (St. Louis, MO, USA). Optical rotations were measured using a JASCO DIP-3000 polarimeter. UV spectra were recorded on a Hitachi U-3210 spectrophotometer. IR spectra were measured by a Perkin-Elmer 100 spectrometer. NMR spectra were obtained on a Bruker AVANCE 500 spectrometer in DMSO-*d*_6_ and referenced to the residual solvent signals (δ_H_ 3.36, δ_C_ 40.6). HRESITOFMS were recorded on a Bruker microTOF apparatus. Cosmosil 75C18-PREP (Nacalai Tesque, Inc., Nakagyo-ku, Kyoto, Japan, 75 μm) was used for ODS column chromatography. HPLC separation was performed using COSMOSIL 5C18-AR-II Packed Column (Nacalai Tesque, Inc., Nakagyo-ku, Kyoto, Japan, 10 × 250 mm) with a photodiode array detector.

### 3.2. Microorganism

Strain MB-PO13 was selected by screening the HAase inhibitory activity of approximately 1000 strains of marine organisms-derived actinomycetes. The strain was isolated from a sea squirt specimem (*Molgula manhattensis*) collected at a harbor near Minato-ku, Tokyo. The strain was identified as a member of genus *Streptomyces* on the basis of 99.2% 16S rRNA gene sequence identity (1429 nucleotides; DDBJ accession number AB840588) with *Streptomyces misawanensis* strain NBRC 13855 (accession number AB184533).

### 3.3. Fermentation

Strain MB-PO13 growing on a yeast-starch agar medium consisting of soluble starch (Wako Pure Chemical Industries, Ltd., Chuo-ku, Osaka, Japan) 1.0%, yeast extract (Becton, Dickinson and Company, Sparks, MD, USA) 0.2%, and agar 1.5% (pH 7.2) was inoculated into 500 mL K-1 flasks each containing 100 mL of the V-22 seed medium consisting of soluble starch 1.0%, glucose 0.5%, NZ-case (Wako Pure Chemical Industries, Ltd., Chuo-ku, Osaka, Japan) 0.3%, yeast extract (Difco Laboratories) 0.2%, tryptone (Difco Laboratories) 0.5%, K_2_HPO_4_ 0.1%, MgSO_4_ 7H_2_O 0.05%, and CaCO_3_ 0.3% (pH 7.0). The flasks were placed on a rotary shaker (200 rpm) at 30 °C for four days. Then, the seed culture (3 mL) was transferred into 500 mL K-1 flasks each containing 100 mL of the A-3 M production medium consisting of soluble starch 2.0%, glycerol 2.0%, glucose 0.5%, Pharmamedia 1.5%, yeast extract 0.3%, and Diaion HP-20 resin (Mitsubishi Chemical Co., Chiyoda-ku, Tokyo, Japan) 1%. The pH of the medium was adjusted to 7.0 before sterilization. The inoculated flasks were placed on a rotary shaker (200 rpm) at 30 °C for seven days.

### 3.4. Extraction and Isolation

After incubation, 100 mL of ethyl acetate was added to each flask, and the flasks were allowed to shake for one hour. The mixture was centrifuged at 6000 rpm for 10 min and the organic layer was separated from the aqueous layer containing the mycelium and evaporated to give 450 mg of crude extract from 1 L of culture. This extract was subjected to reversed-phase ODS column chromatography with a gradient of MeCN/0.1% aqueous HCO_2_H (2:8, 3:7, 4:6, 5:5, 6:4, 7:3, and 8:2 v/v). The fraction eluted with 70% MeCN was pooled and evaporated *in vacuo*, and the major part of the solvent evaporated in vacuum. The remaining aqueous phase was extracted twice with EtOAc concentrated to give a red solid (46 mg). The final purification was achieved by preparative HPLC using a linear gradient of MeCN/0.1% aqueous HCO_2_H (MeCN concentration: 15%–85% for 0–30 min) at 4 mL/min, yielding hyaluromycin (20 mg) with a retention time of 22.5 min.

### 3.5. Hyaluromycin (**1**)

Red powder; [α]^25^_D_ −168 (*c* 0.005, DMSO); UV (1% DMSO in MeOH) λ_max_ (log ε) 257 (4.74), 307 (sh, 4.30), 352 (4.40), 368 (sh, 3.98), 472 (sh, 3.80), 506 (3.78), 544 (sh, 3.62); (1% DMSO in 0.01 N methanolic HCl) 250 (4.60), 307 (sh, 4.22), 354 (4.02), 366 (sh, 3.68), 474 (sh, 3.66), 504 (3.69), 545 (sh, 3.43); (1% DMSO in 0.01 N methanolic NaOH) 236 (4.60), 259 (4.50), 333 (sh, 3.92), 392 (3.94), 503 (sh, 3.77), 539 (3.98), 570 (3.95); IR (ATR) ν_max_ 3357, 2935, 1981, 1693, 1599, 1537, 1440, 1331, 1221 cm^−1^; ^1^H and ^13^C NMR data, see Table 1 and Supplementary Information; HRESITOFMS [M + H]^+^ 604.1091 (calcd for C_30_H_22_NO_13_, 604.1086).

### 3.6. Feeding Experiment

[1,2-^13^C_2_]Acetate-labeled hyaluromycin (**1**) was prepared by culturing the producing strain in a liquid medium containing sodium [1,2-^13^C_2_]acetate. The inoculation, cultivation, extraction and purification were conducted in the same manner as described above. Sodium [1,2-^13^C_2_]acetate (20 mg/mL in distilled water) was added at 48 h after inoculation, then every 24 h four times. After further incubation for two days, the culture broth was extracted with EtOAc. From 1 L culture, 20 mg of [1,2-^13^C_2_]acetate-labeled **1** was obtained.

### 3.7. Methylation of [1,2-^13^C_2_]Acetate-Labeled Hyaluromycin (**1**)

3″-*O*-Methylhyaluromycin (**2**) labeled with [1,2-^13^C_2_]acetate: DBU (40 μL, 0.27 μmol) and CH_3_I (400 μL, 6.43 μmol) were added to a stirred solution of [1,2-^13^C_2_]acetate-labeled **1** (20.0 mg, 0.83 μmol) in Me_2_CO/MeCN (400 μL each). After heating at 50 °C for 1 h, the reaction mixture was diluted with water and EtOAc (500 μL each), and the organic layer was separated and evaporated *in vacuo*. The residue was purified by ODS column chromatography by a gradient of MeCN/0.1% aqueous HCO_2_H (2:8, 3:7, 4:6, 5:5, 6:4, 7:3, and 8:2 v/v). Final purification was achieved by preparative HPLC using a linear gradient of MeCN/0.1% aqueous HCO_2_H (MeCN concentration: 15%–85% for 0–30 min) at 4 mL/min to give 3″-*O*-methyl [1,2-^13^C_2_]acetate-labeled hyaluromycin (**2**, 7.2 mg, 36% yield, *t*_R_ = 18.8 min) as a red powder. For physico-chemical properties, see the data for non-labeled **2** described below. ^1^H and ^13^C NMR data, see Table 1 and Supplementary Information.

### 3.8. Methylation of Hyaluromycin (**1**)

For the measurement of physico-chemical properties and biological evaluation, a small portion of non-labeled **1** was methylated to give non-labeled **2** in the same manner as described above. 3″-*O*-methylhyaluromycin (**2**): Red powder; [α]^25^_D_ −77 (*c* 0.005, DMSO); UV (1% DMSO in MeOH) λ_max_ (log ε) 249 (4.82), 314 (sh, 4.02), 350 (4.10), 366 (sh, 3.77), 498 (3.88); (1% DMSO in 0.01 N methanolic HCl) 248 (4.75), 315 (sh, 4.30), 356 (4.15), 491 (3.87), 520 (3.77); (1% DMSO in 0.01 N methanolic NaOH) 254 (4.50), 392 (3.81), 538 (3.90), 560 (3.89); IR (ATR) ν_max_ 3348, 2934, 1677, 1604, 1514, 1439, 1331, 1228 cm^−1^; ^1^H and ^13^C NMR data, see Supplementary Information; HRESITOFMS [M + Na]^+^ 640.1057 (calcd for C_31_H_24_NO_13_Na, 640.1062).

### 3.9. Hyaluronidase Inhibitory Activity

HAase inhibitory activity was measured by the turbidimetric assay described by Ferrante [63] with slight modifications. HAase (EC 3.2.1.35) from the bovine testes type I-S (Sigma-Aldrich Co. LCC, St. Louis, MO, USA) and HA sodium salt from rooster comb (Wako Pure Chemical Industries, Ltd., Chuo-ku, Osaka, Japan) were dissolved in acetate buffer (0.2 M sodium acetate, 0.15 M NaCl, pH 5.0). The mixtures contained 100 μL of 0.01% HAase and 20 μL of either 0.0031%–0.10% **1**, 0.013%–0.50% **2**, 0.013%–0.50% β-rubromycin (isolated from *Streptomyces*), 0.013%–0.50% γ-rubromycin (BioViotica Naturstoffe GmbH, Dransfelder Weg, Dransfeld, Germany) or 0.050%–2.0% glycyrrhizin (Tokyo Chemical Industry Co., Ltd., Chuo-ku, Tokyo, Japan) in DMSO (Table 2). The mixtures were incubated at 37 °C for 20 min. After incubation, 100 μL of 0.1% HA was added and the mixtures were further incubated at 37 °C for 60 min. After incubation, the enzymatic reaction was terminated by the addition of 1 mL of 2.5% cetyltrimethylammonium bromide (CTAB) in 2% aqueous NaOH. The turbidity at 400 nm was measured after 30 min. All incubations were performed in triplicate.

## 4. Conclusions

Hyaluromycin (**1**), a new member of rubromycin family of antibiotics, was isolated from a marine-derived *Streptomyces* sp. as a HAase inhibitor on the basis of HAase activity screening. Hyaluromycin (**1**) consists of rubromycin common structure and 2-amino-3-hydroxycyclopent-2-enone (C_5_N) structure; both structures units have been reported only from actinomycetes. Hyaluromycin (**1**) displayed approximately 25-fold more potent inhibitory activity against HAase than did glycyrrhizin, a well-known plant terpenoid. Interestingly, β-rubromycin (**4**) and γ-rubromycin (**5**), lacking the C_5_N unit, showed no inhibitory activity. More noteworthy is that the derivative **2** in which the enol hydroxyl group in the cyclopentane unit is protected as a methyl ether showed no inhibitory activity. These results suggest that the C_5_N unit plays an essential role in the observed hyaluronidase inhibition. The present study may provide new insight for developing new, promising anti-inflammation molecules.

## Supplementary Files

Supplementary File 1 Supplementary Information (PDF, 2324 KB)
