# Supplementary material for: Hyaluromycin, a New Hyaluronidase Inhibitor of Polyketide Origin from Marine Streptomyces sp
Source: Mar Drugs. 2014 Jan 21;12(1):491–507. doi: 10.3390/md12010491 (PMC3917283; doi:10.3390/md12010491)
Supplement: Supplementary File 1 — Supplementary Information (PDF, 2324 KB) [file marinedrugs-12-00491-s001.pdf]

Supplementary Information

Figure S1. ^{1}H NMR spectrum of hyaluromycin (**1**) (500 MHz, DMSO-d_6).
Figure S2. ^{13}C NMR spectrum of **1** (125 MHz, DMSO-d_6).
Figure S3. ^{1}H-^{1}H COSY spectrum of **1** (500 MHz, DMSO-d_6).
Figure S4. HSQC spectrum of **1** (500 MHz, DMSO-d_6).
Figure S5. HMBC spectrum of **1** (500 MHz, DMSO-d_6).
Figure S6. ^{1}H NMR spectrum of 3″-*O*-methyl hyaluromycin (**2**) (500 MHz, DMSO-d_6).
Figure S7. ^{13}C NMR spectrum of **2** (125 MHz, DMSO-d_6).
Figure S8. HSQC spectrum of **2** (500 MHz, DMSO-d_6).
Figure S9. HMBC spectrum of **2** (500 MHz, DMSO-d_6).
Figure S10. ^{13}C NMR spectrum of [1,2-$^{13}C_2$]acetate-labeled **1** (500 MHz, DMSO-d_6).
Figure S11. ^{1}H NMR spectrum of [1,2-$^{13}C_2$]acetate-labeled **2** (500 MHz, DMSO-d_6).
Figure S12. ^{13}C NMR spectrum of [1,2-$^{13}C_2$]acetate-labeled **2** (125 MHz, DMSO-d_6).
Figure S13. ^{1}H-^{1}H COSY spectrum of [1,2-$^{13}C_2$]acetate-labeled **2** (500 MHz, DMSO-d_6).
Figure S14. HSQC spectrum of [1,2-$^{13}C_2$]acetate-labeled **2** (500 MHz, DMSO-d_6).
Figure S15. HMBC spectrum of [1,2-$^{13}C_2$]acetate-labeled **2** (500 MHz, DMSO-d_6).
Figure S16. INADEQUATE spectrum of [1,2-$^{13}C_2$]acetate-labeled **2** (125 MHz, DMSO-d_6, $^1J_{C\text{-}C}$ = 50 Hz).
Figure S17. INADEQUATE spectrum of [1,2-$^{13}C_2$]acetate-labeled **2** (125 MHz, DMSO-d_6, $^1J_{C\text{-}C}$ = 35 Hz).

Figure S1. ^{1}H NMR spectrum of hyaluromycin (**1**) (500 MHz, DMSO-d_6).

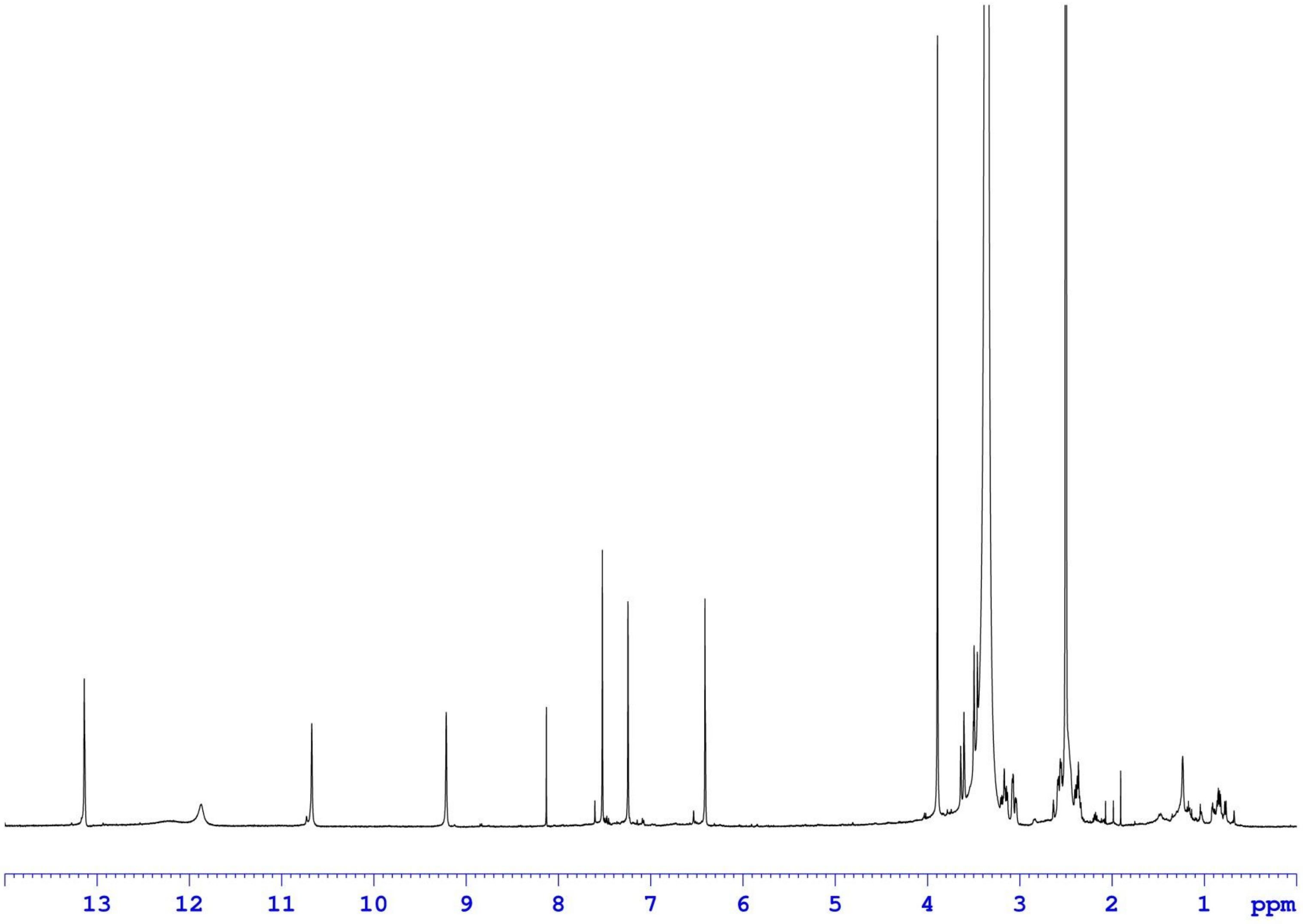

Figure S2. ^{13}C NMR spectrum of **1** (125 MHz, DMSO-d_6).

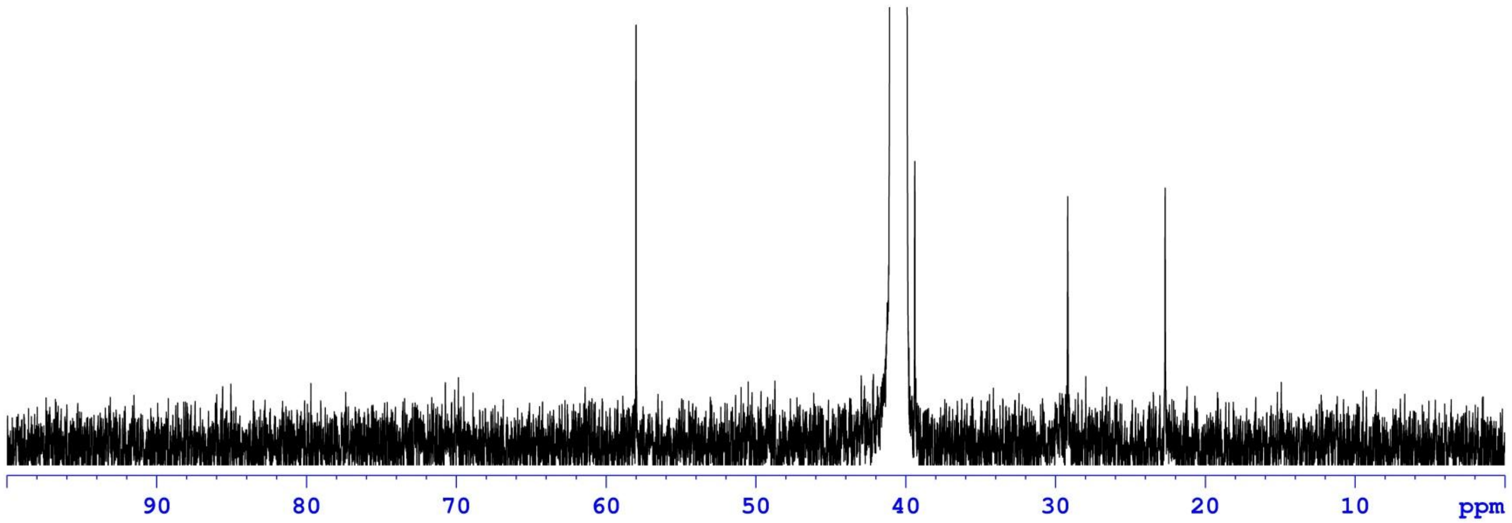

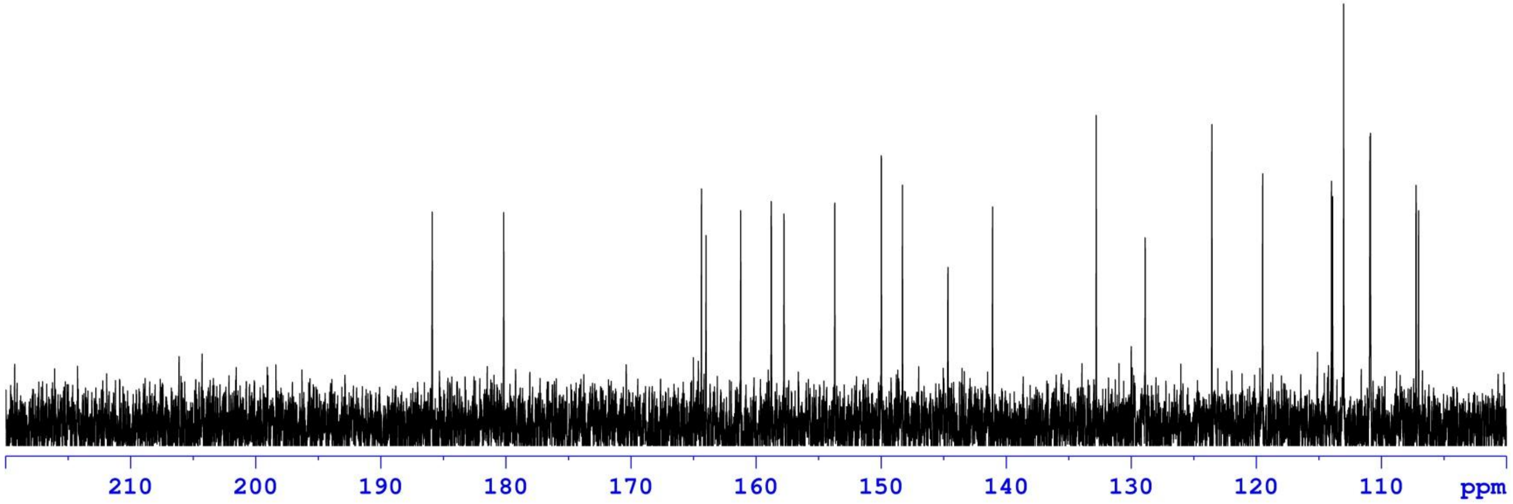

Figure S3. ^{1}H-^{1}H COSY spectrum of **1** (500 MHz, DMSO-d_6).

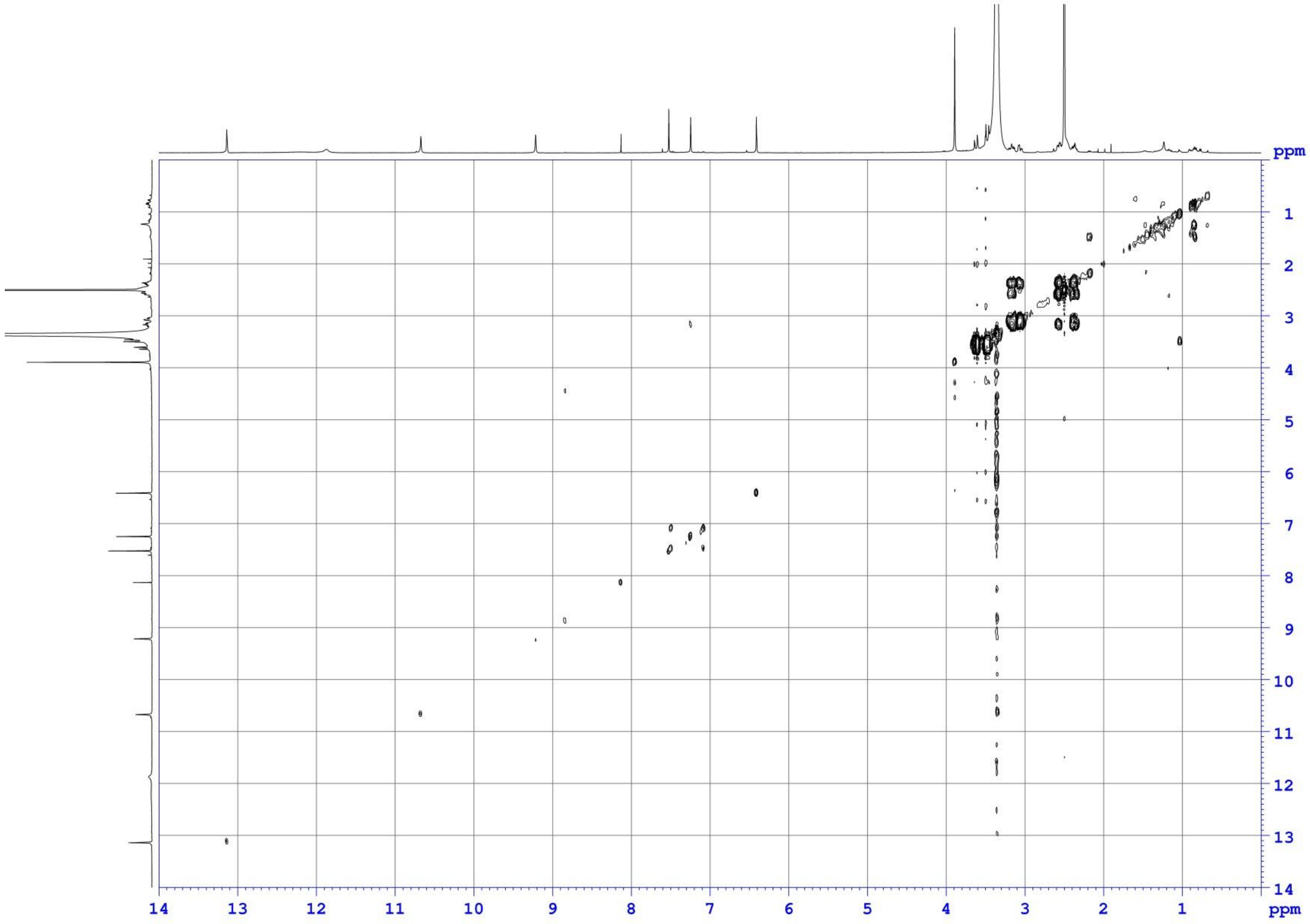

Figure S4. HSQC spectrum of **1** (500 MHz, DMSO-d_6).

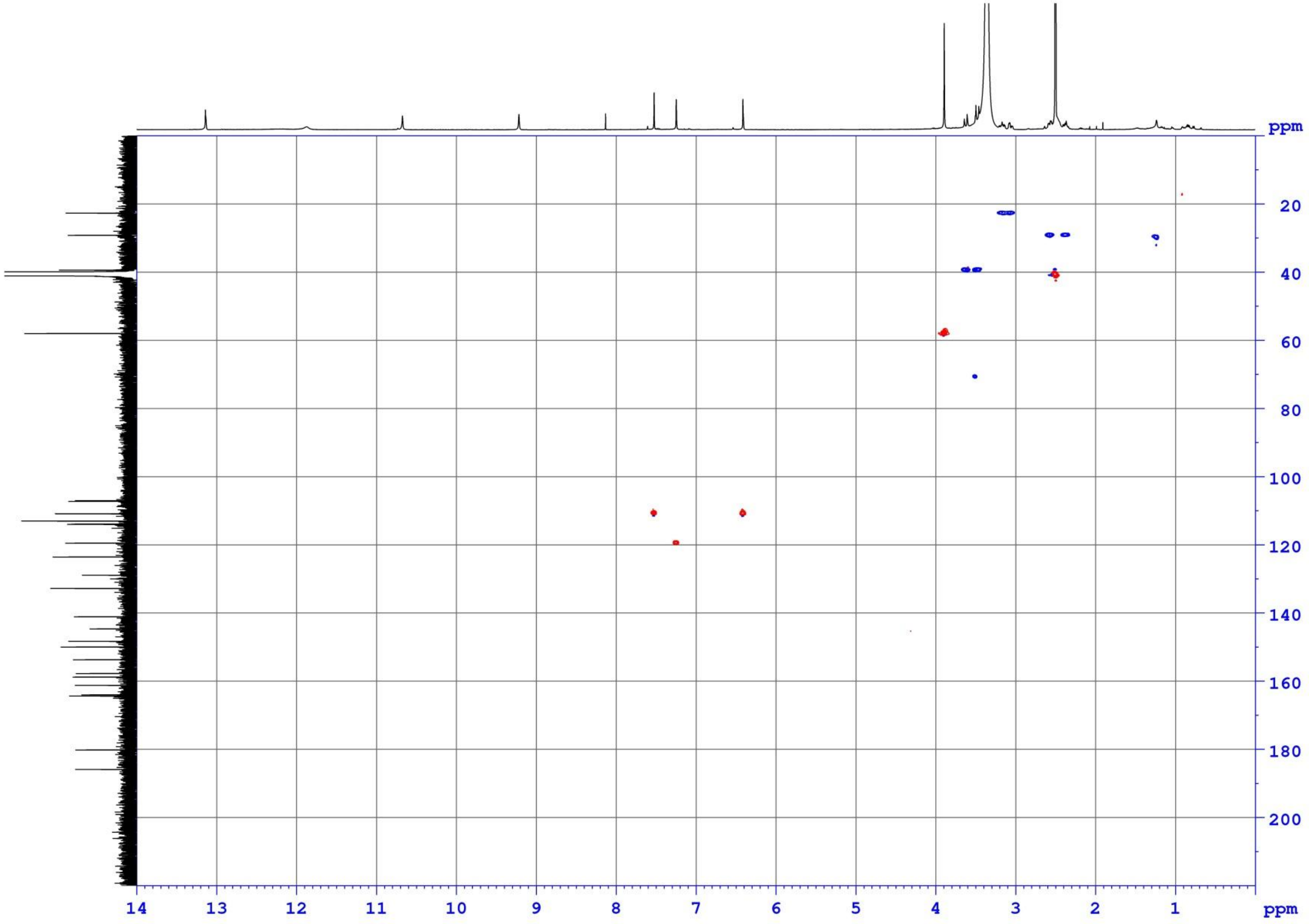

Figure S5. HMBC spectrum of **1** (500 MHz, DMSO-d_6).

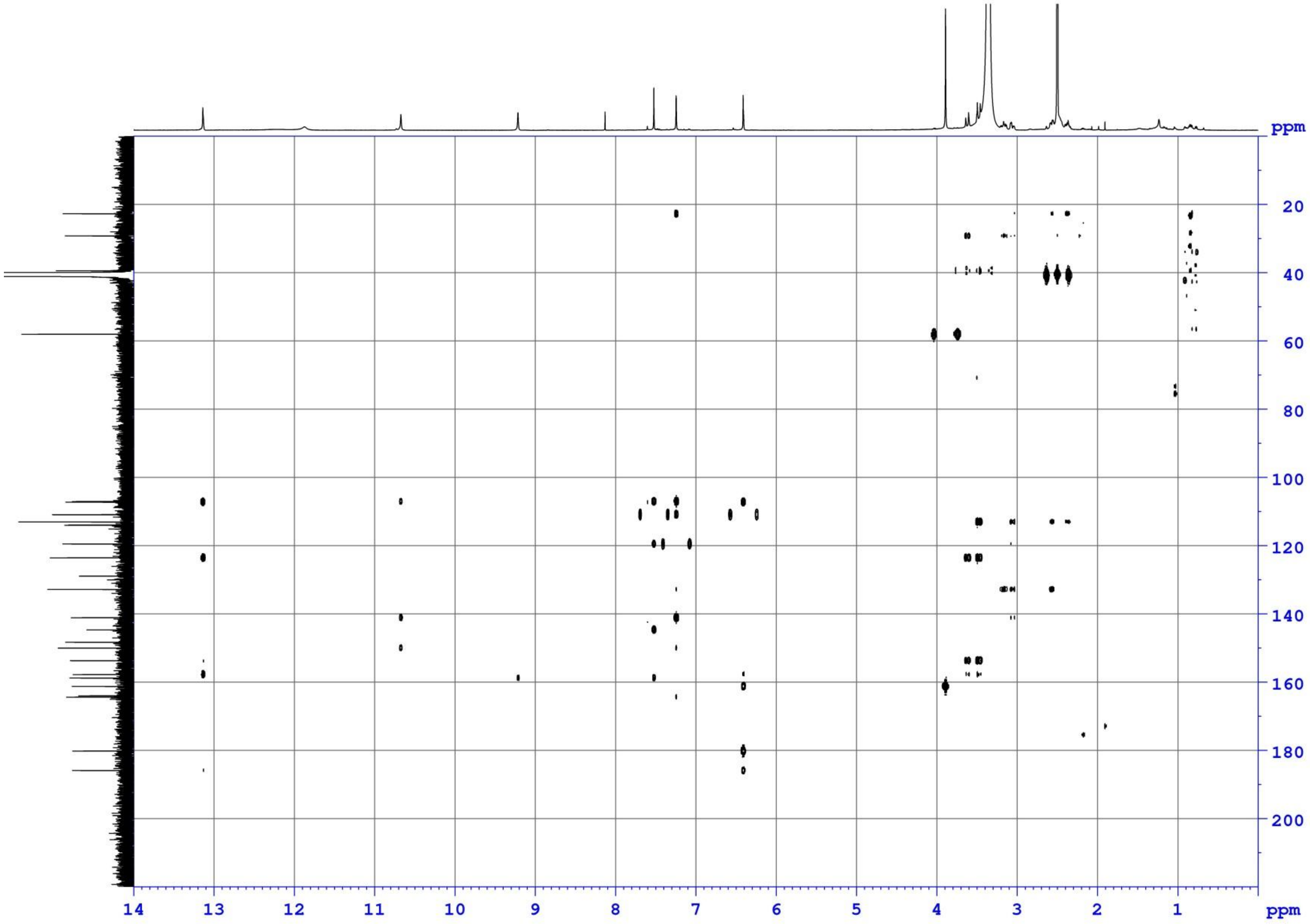

Figure S6. ^{1}H NMR of 3″-*O*-methyl hyaluromycin (**2**) (500 MHz, DMSO-d_6).

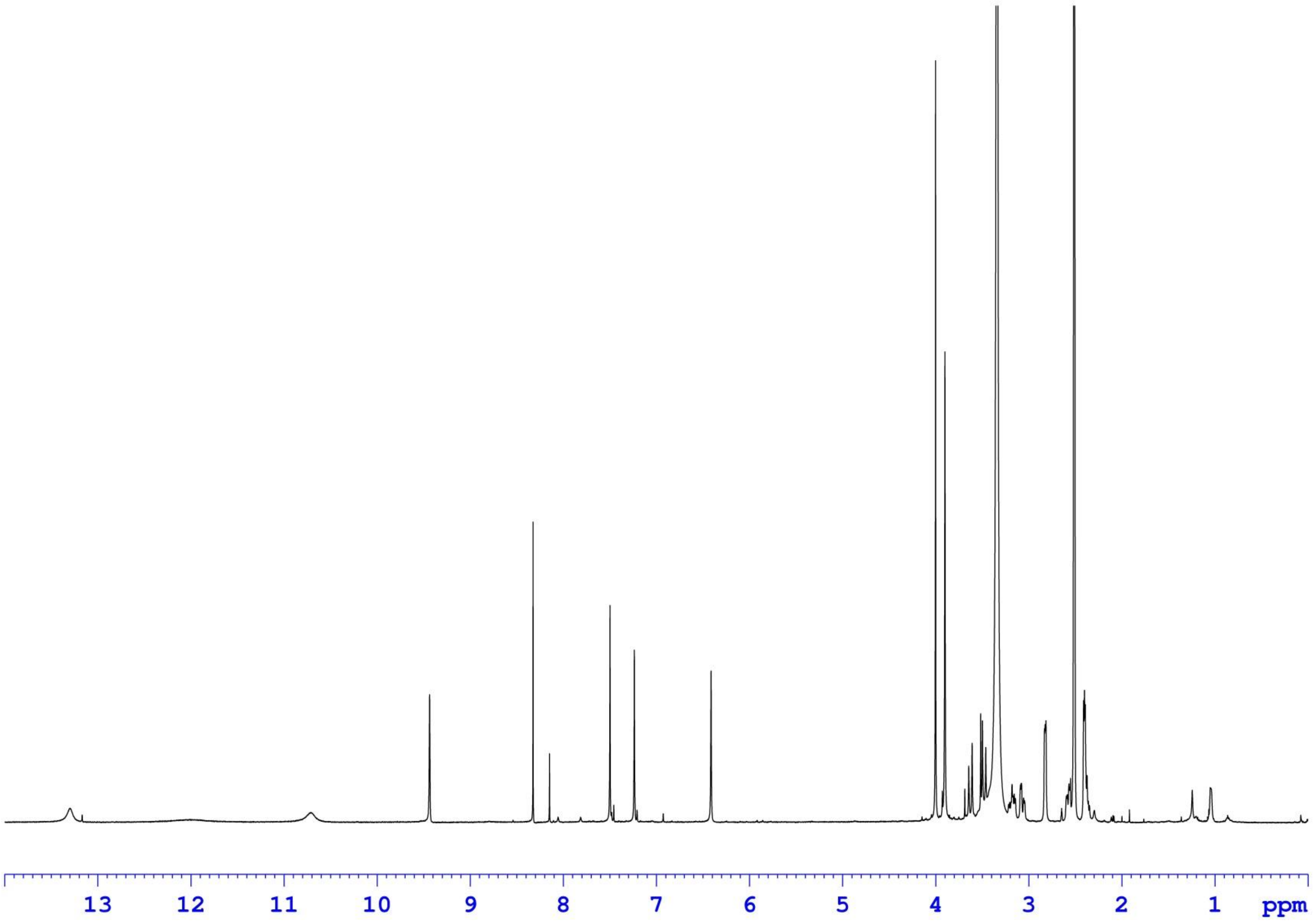

Figure S7. ^{13}C NMR spectrum of **2** (125 MHz, DMSO-d_6).

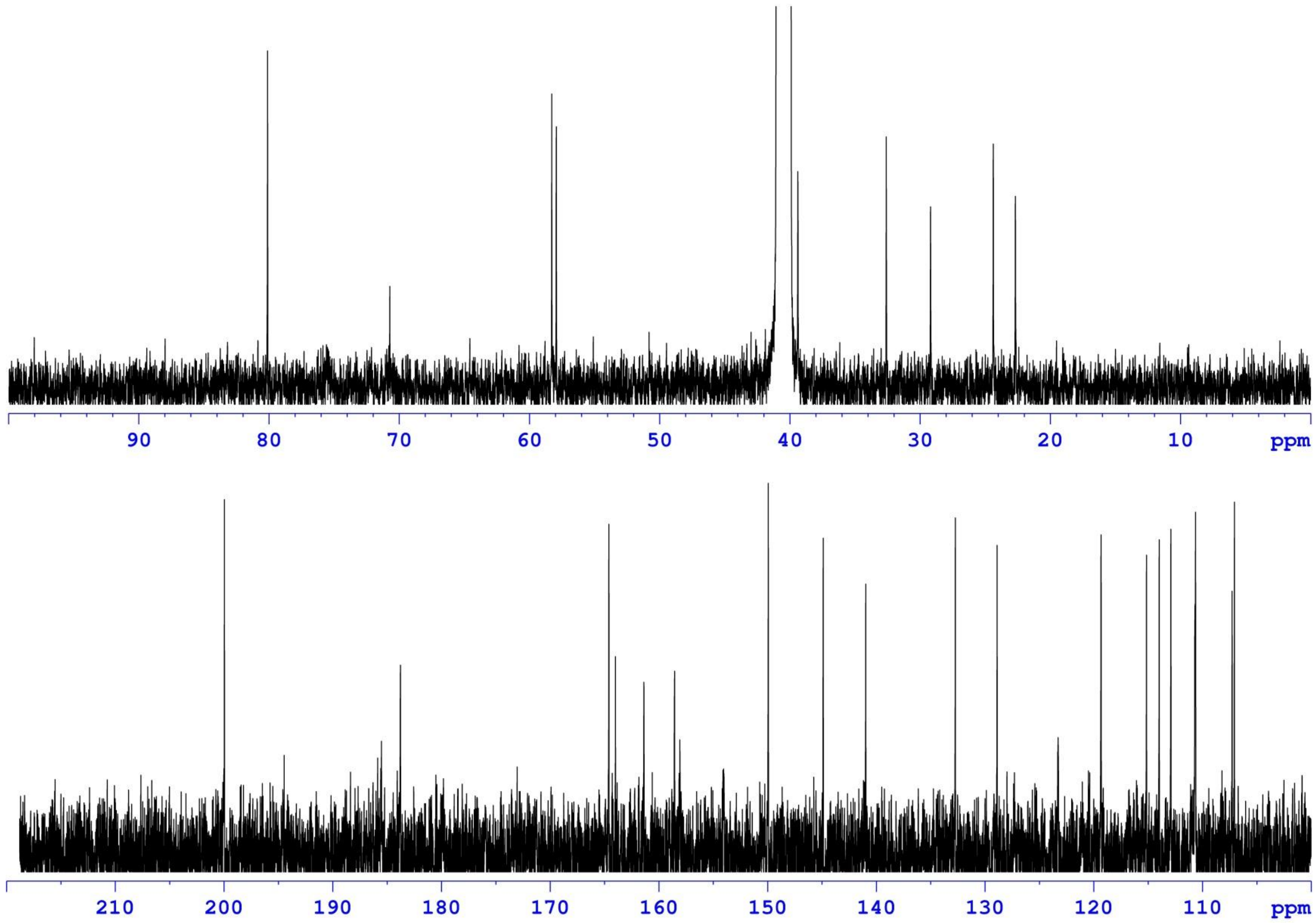

Figure S8. HSQC spectrum of **2** (500 MHz, DMSO-d_6).

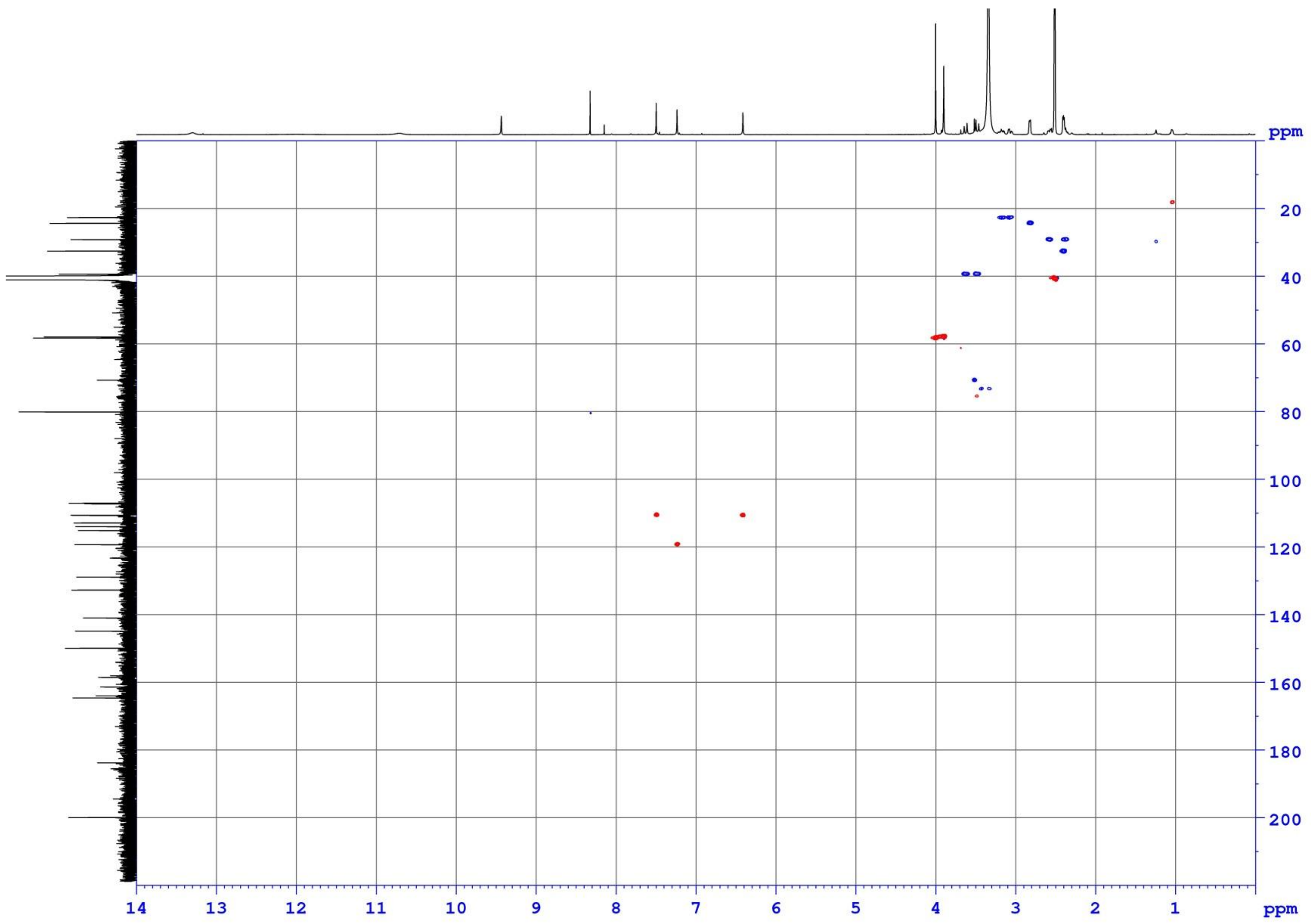

Figure S9. HMBC spectrum of **2** (500 MHz, DMSO-d_6).

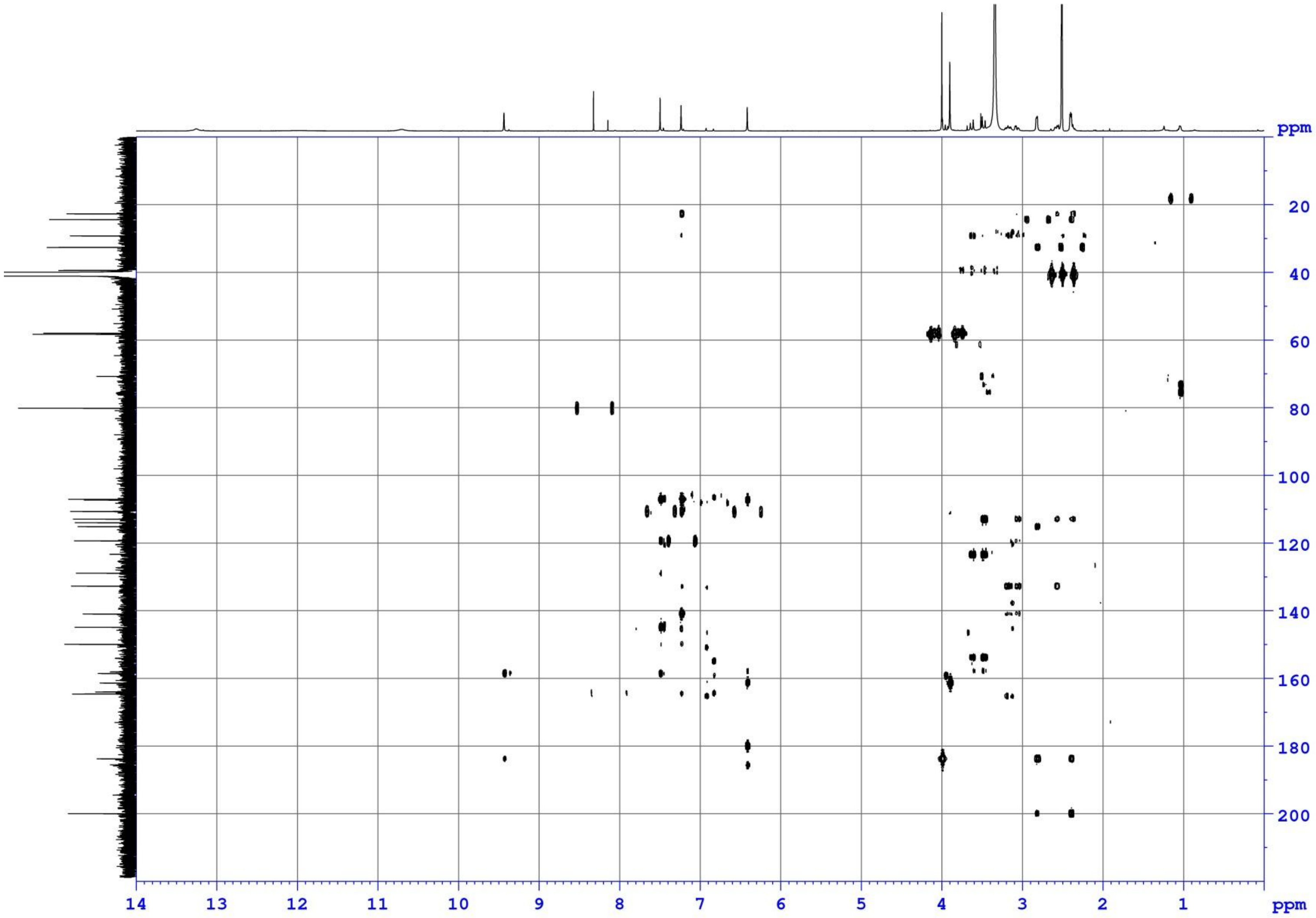

Figure S10. ^{13}C NMR spectrum of [1,2-$^{13}C_2$]acetate-labeled **1** (500 MHz, DMSO-d_6).

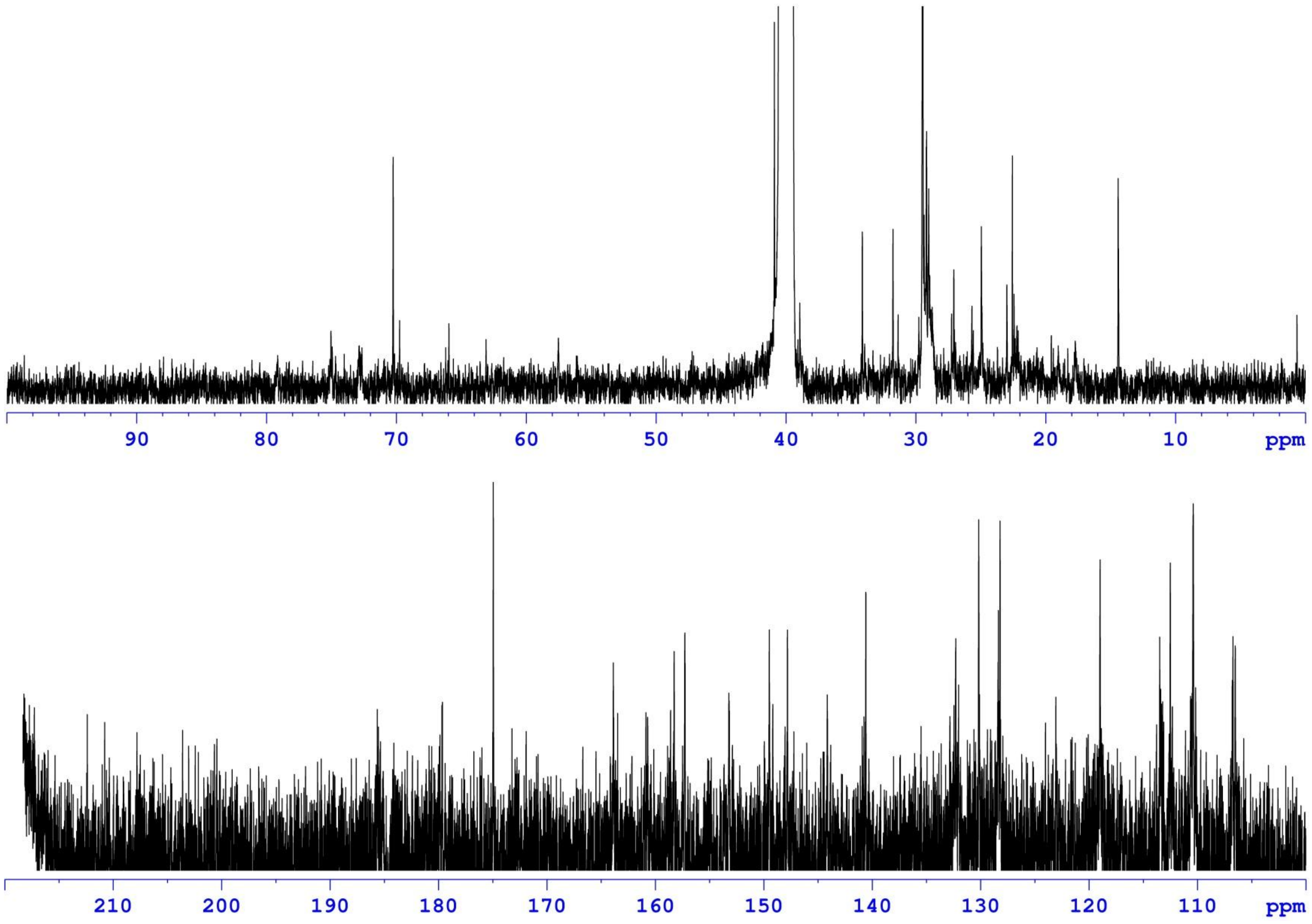

Figure S11. ^{1}H NMR of [1,2-$^{13}C_2$]acetate-labeled **2** (500 MHz, DMSO-d_6).

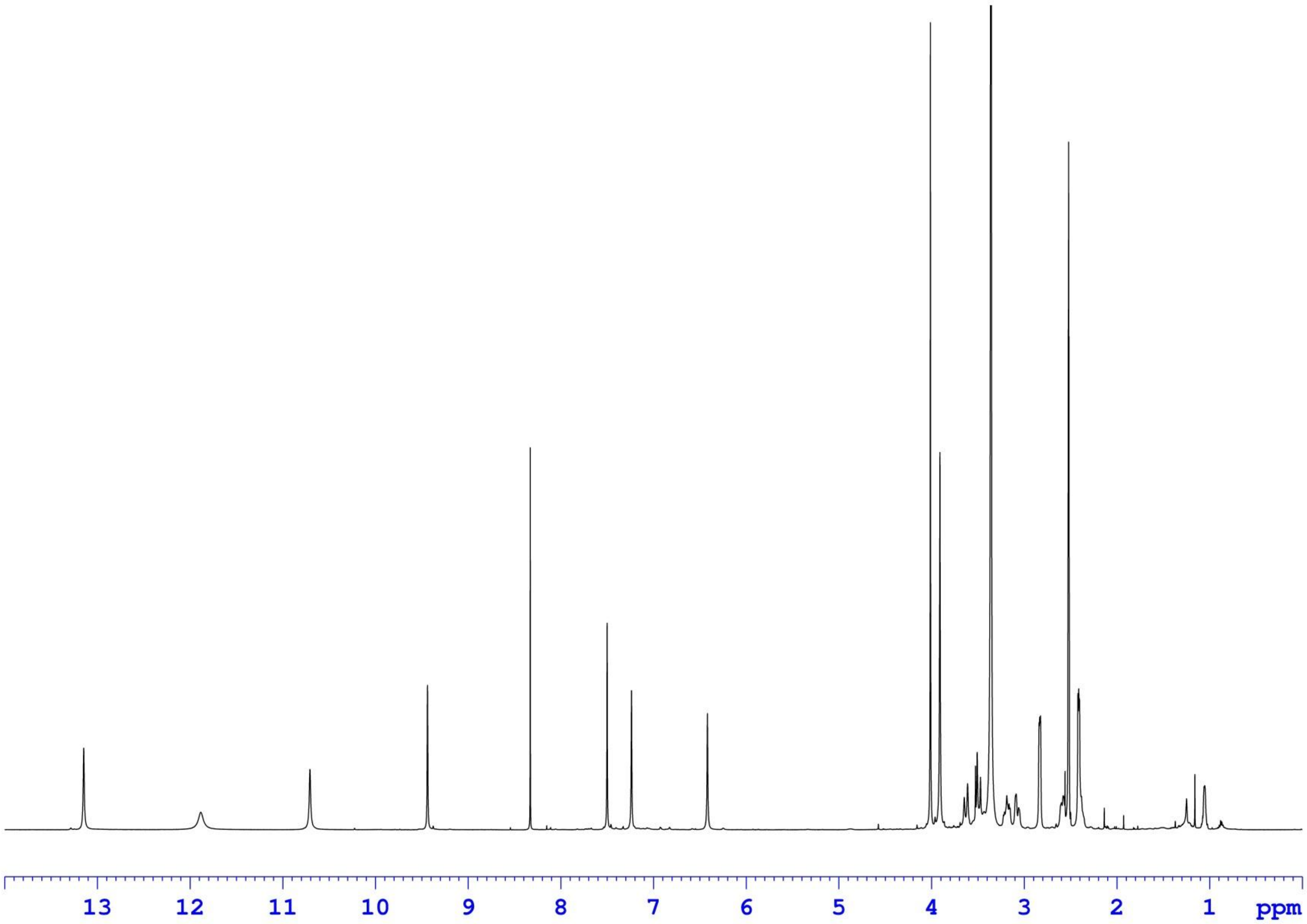

Figure S12. ^{13}C NMR spectrum of [1,2-$^{13}C_2$]acetate-labeled **2** (125 MHz, DMSO-d_6).

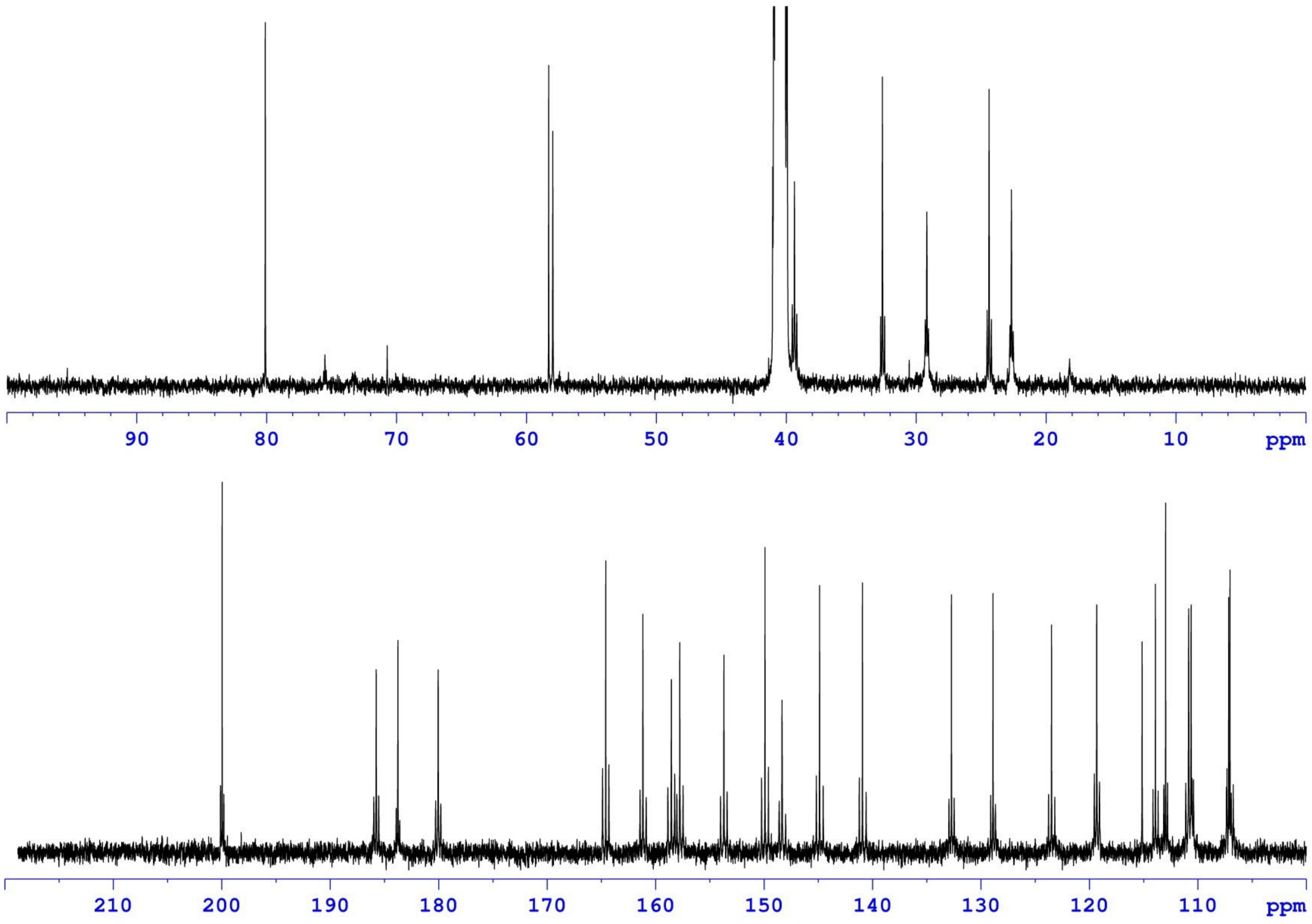

Figure S13. ^{1}H-^{1}H COSY spectrum of [1,2-$^{13}C_2$]acetate-labeled **2** (500 MHz, DMSO-d_6).

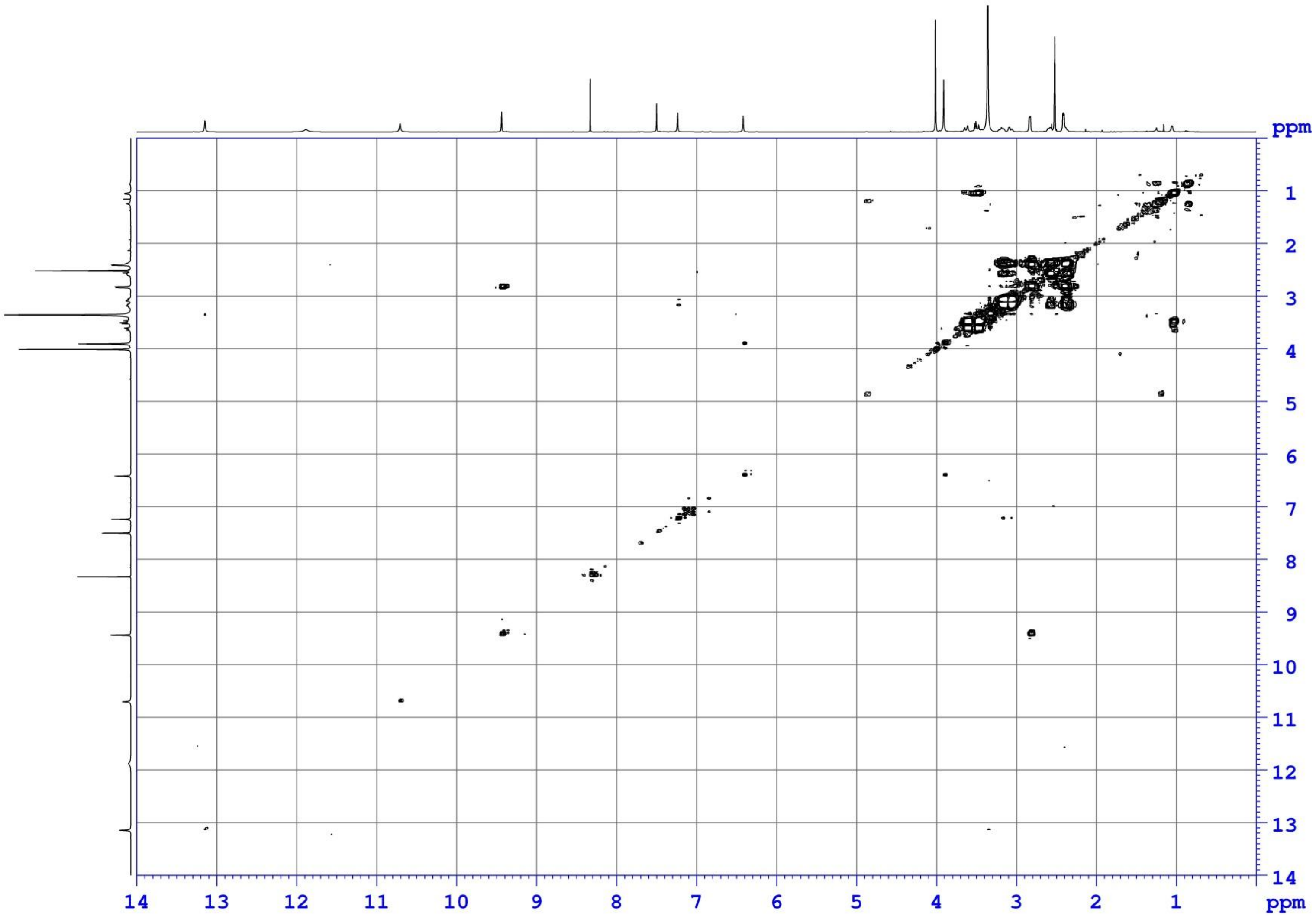

Figure S14. HSQC spectrum of [1,2-$^{13}C_2$]acetate-labeled **2** (500 MHz, DMSO-d_6).

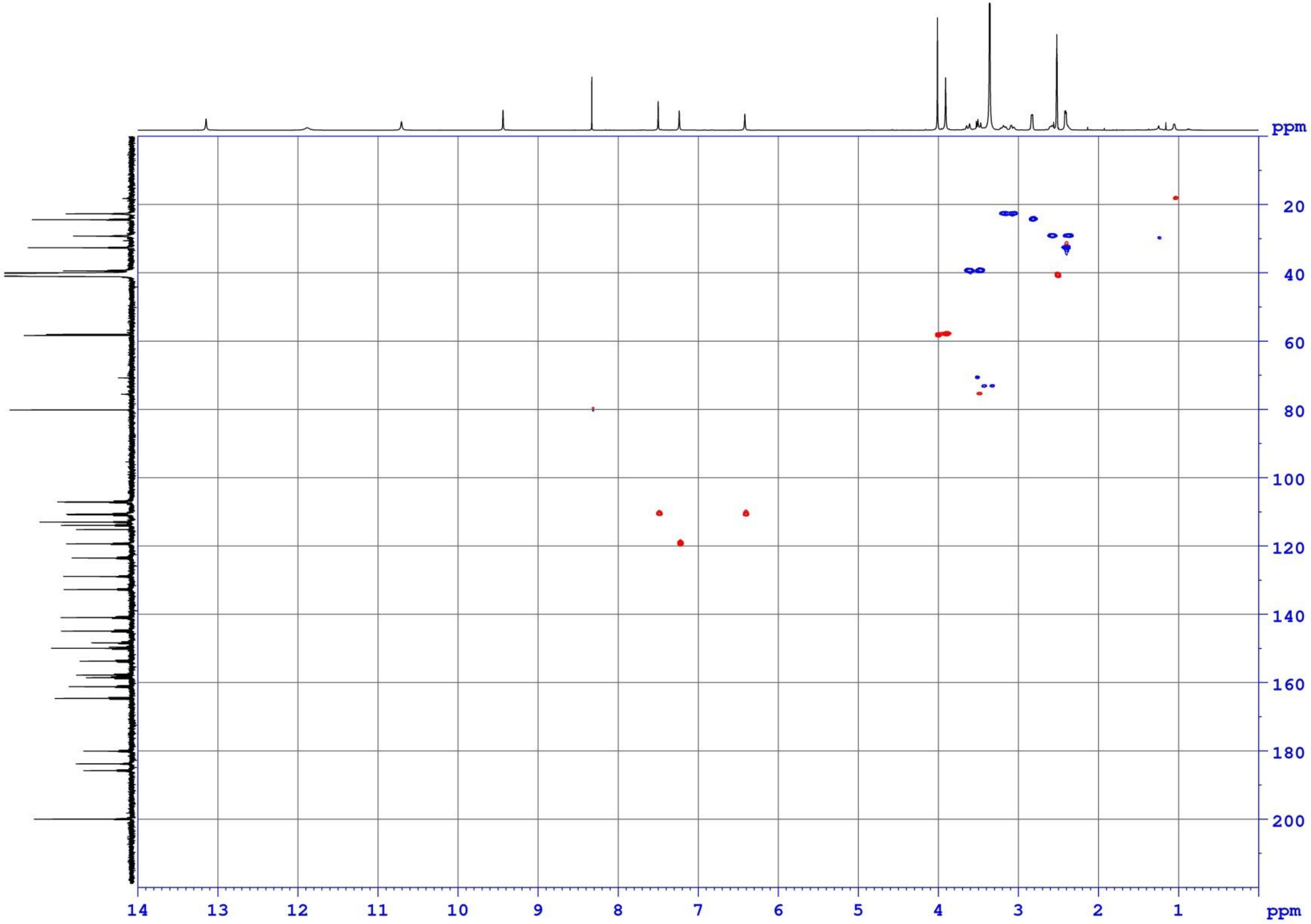

Figure S15. HMBC spectrum of [1,2-$^{13}C_2$]acetate-labeled **2** (500 MHz, DMSO-d_6).

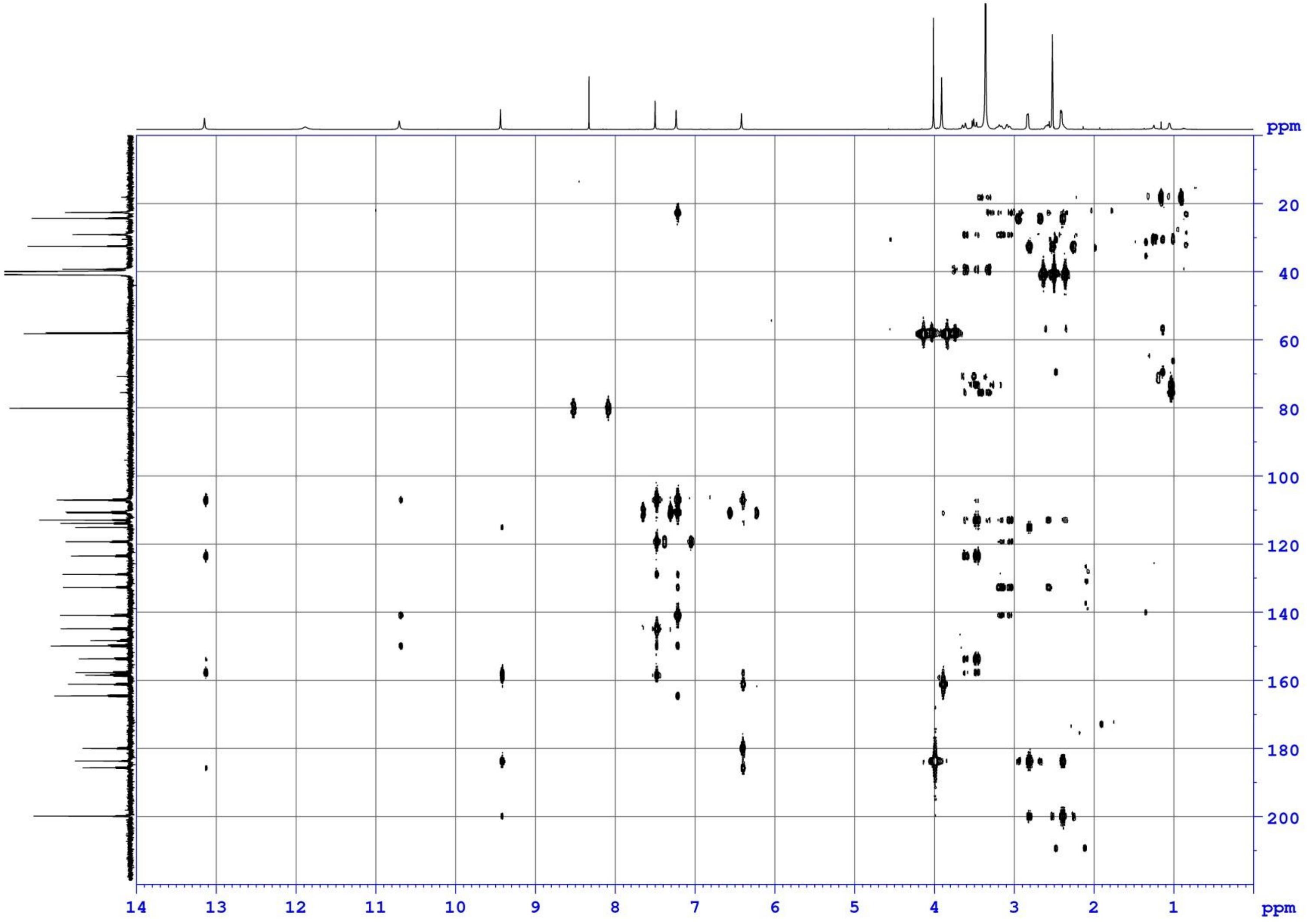

Figure S16. INADEQUATE spectrum of [1,2-$^{13}C_2$]acetate-labeled **2** (125 MHz, DMSO-d_6, $^1J_{C\text{-}C}$ = 50 Hz).

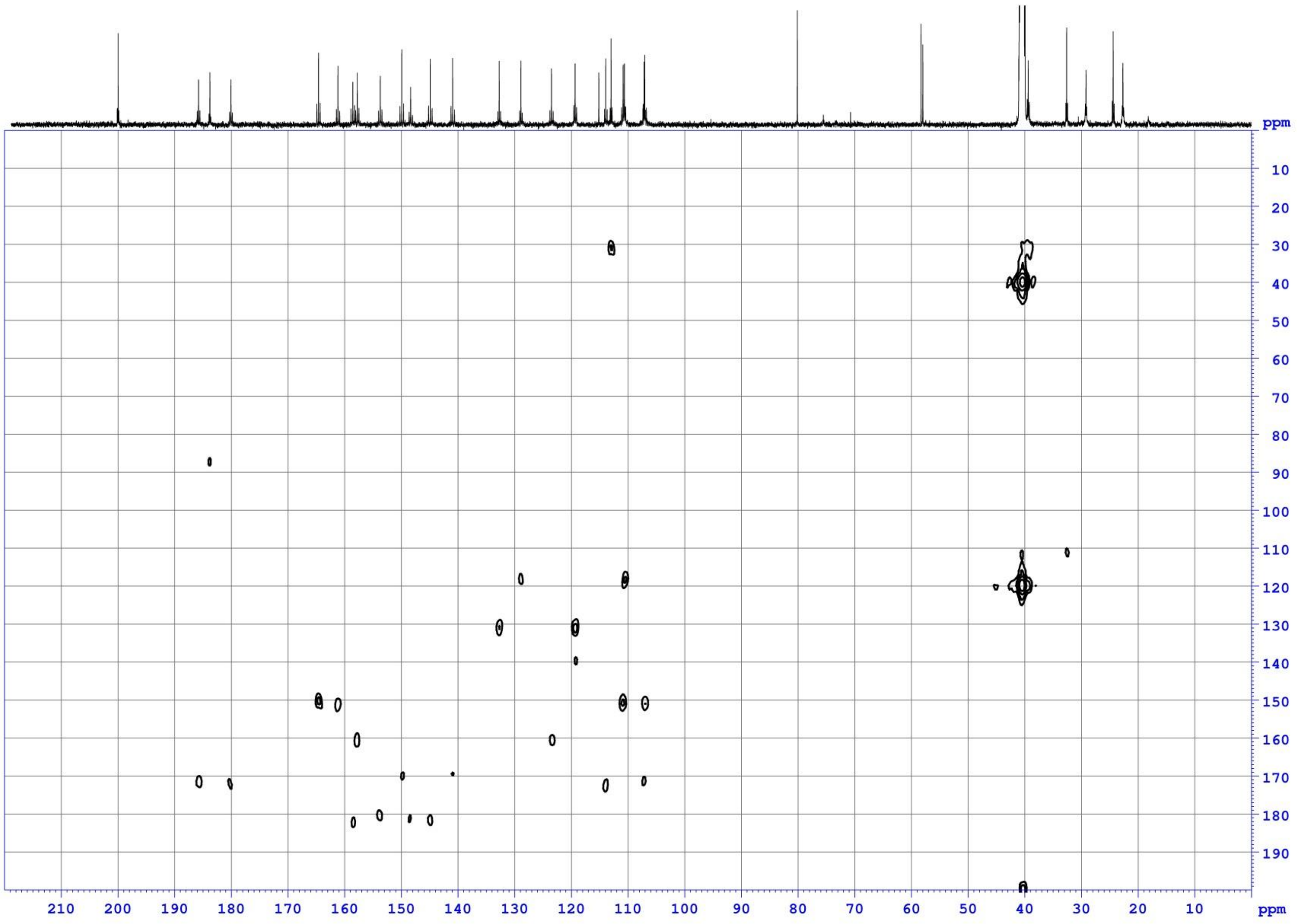

Figure S17. INADEQUATE spectrum of [1,2-$^{13}C_2$]acetate-labeled **2** (125 MHz, DMSO-d_6, $^1J_{C\text{-}C}$ = 35 Hz).

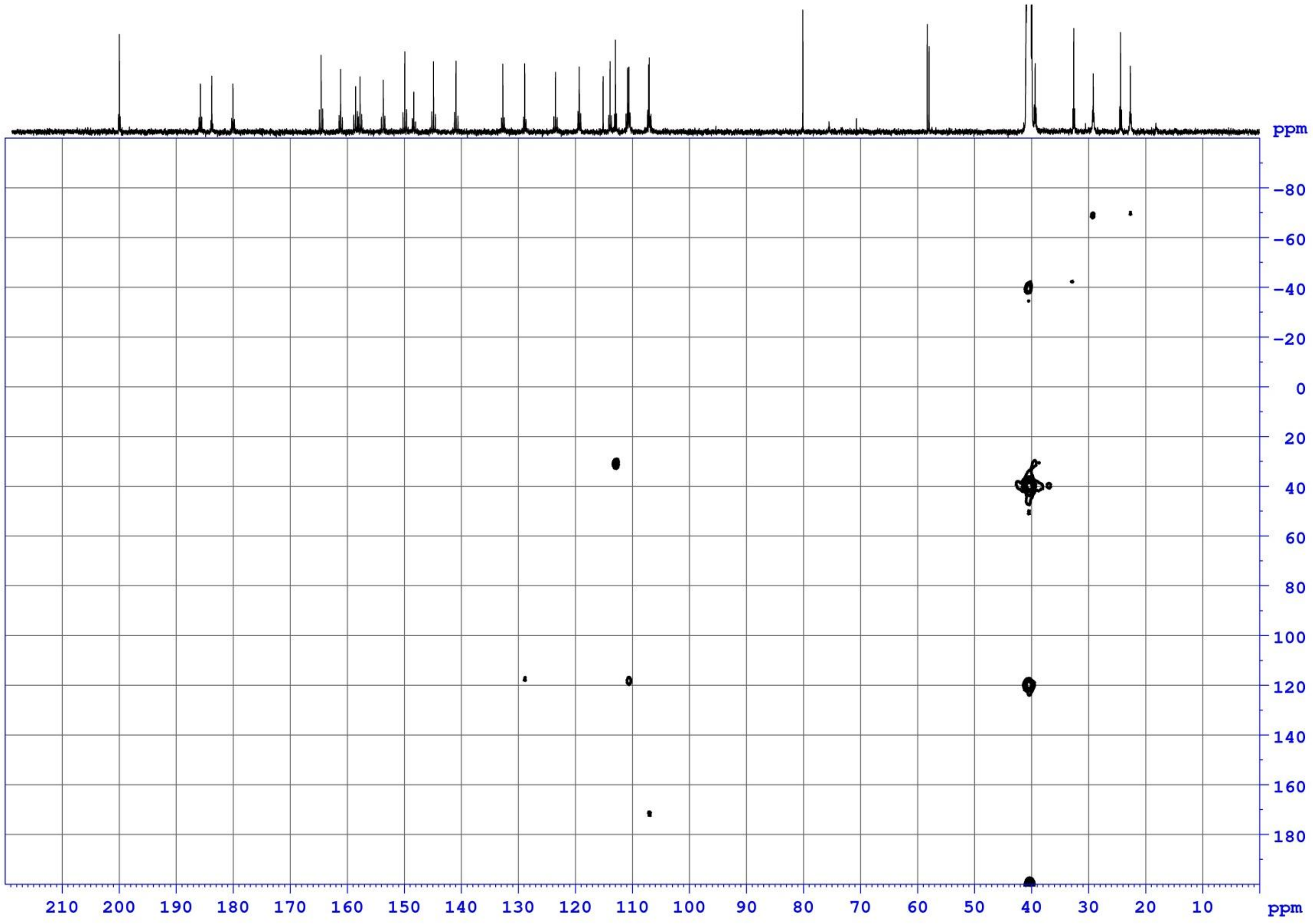

© 2014 by the authors; licensee MDPI, Basel, Switzerland. This article is an open access article distributed under the terms and conditions of the Creative Commons Attribution license (http://creativecommons.org/licenses/by/3.0/).
